# Are therapeutic effects of antiacne agents mediated by activation of FoxO1 and inhibition of mTORC1?

**DOI:** 10.1111/exd.12172

**Published:** 2013-06-25

**Authors:** Bodo C Melnik, Gerd Schmitz

**Affiliations:** 1 Department of Dermatology, Environmental Medicine and Health Theory, University of OsnabrückOsnabrück, Germany; 2 Institute for Clinical Chemistry and Laboratory Medicine, University Hospital Regensburg, University of RegensburgRegensburg, Germany

**Keywords:** acne, antiacne drugs, FoxOs, mTORC1, mTORC1 inhibitors

## Abstract

Acne pathogenesis has recently been linked to decreased nuclear FoxO1 levels and increased mTORC1 activity. This hypothesis postulates that antiacne agents either enhance nuclear FoxO activity or inhibit mTORC1. Benzoyl peroxide (BPO), by activation of oxidative stress-inducible kinases, increases nuclear FoxO levels promoting Sestrin3-mediated AMPK activation. Furthermore, BPO-derived ROS may activate AMPK via ataxia–telangiectasia mutated. Isotretinoin and *all-trans* retinoic acid may stimulate FoxO gene expression. Doxycycline may enhance FoxOs nuclear retention by inhibiting the expression of exportin 1. Suppression of TNFα signalling by tetracyclines, erythromycin and other macrolides may attenuate IKKβ-TSC1-mediated mTORC1 activation. Erythromycin attenuates ERK1/2 activity and thereby increases TSC2. Azelaic acid may decrease mTORC1 by inhibiting mitochondrial respiration, increasing cellular ROS and nuclear FoxO levels. Antiandrogens may attenuate mTORC1 by suppressing mTORC2-mediated Akt/TSC2 signalling. This hypothesis unmasks a common mode of action of antiacne agents as either FoxO enhancers or mTORC1 inhibitors and thus provides a rational approach for the development of new antiacne agents.

## Introduction

The recent viewpoint on the pathogenesis of acne 1 focused on decreased nuclear levels of the transcription factor FoxO1 and enhanced activity of the kinase mechanistic (or mammalian) target of rapamycin complex 1 (mTORC1). Notably, FoxOs are critical rheostats that inhibit mTORC1 1,2. The hypothesis presented here suggests that antiacne agents either enhance nuclear FoxO1 activity or directly inhibit mTORC1. Improvement of acne by dietary and pharmacological intervention appears to be related to a common underlying mechanism: enhanced FoxO and attenuated mTORC1 signalling 3,4.

## Benzoyl peroxide

Whereas benzoyl peroxide (BPO)-mediated extinction of *P. acnes* takes only minutes, acne improvement takes weeks of treatment. This points to another *P. acnes*-independent mode of BPO action, which apparently involves reactive oxygen species (ROS)-mediated increases in nuclear FoxO levels by ROS-inducible kinases, Jun-N-terminus kinase (JNK) and STE20-like protein kinase 1 (MST1) 5. Notably, JNK- and MST1-mediated FoxO phosphorylation is dominant to the inhibitory Akt-mediated FoxO phosphorylation 6. Addition of hydrogen peroxide to follicular granulosa cells stimulates FoxO1's nuclear translocation 7.

**Figure 1:**
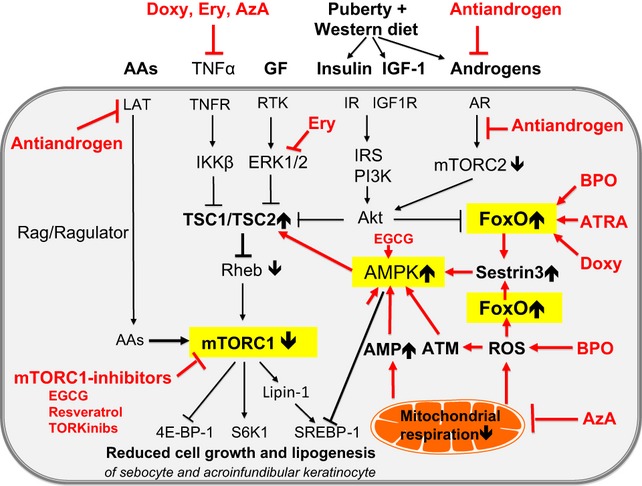
Upregulation of FoxO1 and inhibition of mTORC1 by antiacne agents: oral isotretinoin, *all-trans* retinoic acid (ATRA), doxycycline (Doxy) and benzoyl peroxide (BPO) increase nuclear FoxO levels, which stimulate the expression of Sestrin3. Sestrin3 activates AMPK and augments the inhibitory function of TSC2 towards Rheb, thus suppressing mTORC1. BPO may stimulate ROS-mediated activation of ATM, a further stimulator of AMPK-mediated mTORC1 inhibition. Azelaic acid (AzA) via inhibition of mitochondrial respiration may increase ROS-mediated upregulation of FoxOs and Sestrin3 expression as well as cellular AMP levels, all activating AMPK. Antiandrogens inhibit mTORC2-dependent activation of Akt, thus increasing TSC1/TSC2-mediated inhibition of Rheb. Antiandrogens suppress the expression of L-type amino acid (AA) transporter (LAT), thus interfering with AA-mediated activation of mTORC1. Erythromycin and other macrolides inhibit IKKβ and ERK1/2 signalling and thereby increase the inhibitory activity of the TSC1/TSC2 complex towards Rheb/mTORC1. Natural mTORC1 inhibitors like resveratrol and epigallocatechin-3-gallate (EGCG) as well as synthetic mTOR inhibitors inhibit the ATP-dependent kinase domain of mTOR, thereby directly reducing mTORC1 activity. Thus, all antiacne drugs may directly or indirectly impair downstream mTORC1 signalling and may attenuate growth and proliferation of acroinfundibular keratinocytes, sebocytes and sebaceous lipogenesis.

BPO-mediated FoxO upregulation may attenuate mTORC1, which controls the G1/S transition and G2/M progression of the cell cycle 8. BPO reduces the size of golden hamster ear sebaceous glands (SGs) and the number of sebocytes entering the S-phase 9. Antiproliferative effects of BPO have been confirmed in human SGs 10,11. In the rabbit ear microcomedo prevention assay, BPO decreases size and number of corneocytes and comedo formation 12,13 (Fig. 1).

## Tetracyclines

Inflammatory events are associated with the initiation of acne 14. Tetracyclines exert anti-inflammatory effects. FoxOs are pivotal regulators of inflammation 15,16. FoxO transport through the nuclear pore requires its interaction with importins and exportins 17. Exportin 1, also known as *chromosomal region maintenance protein 1* (CRM1), recognizes specific nuclear export signals (NESs) present on FoxO1 including a leptomycin-sensitive NES 18. Intriguingly, in a mouse lung tumor model, doxycycline dramatically decreases CRM1 expression in comparison with untreated mice 19. Thus, the anti-inflammatory and possibly antiproliferative effect of doxycycline may derive from enhanced nuclear FoxO1 retention.

Tetracyclines inhibit NFκB activation and TNFα secretion 20. IKKβ, the crucial kinase of proinflammatory NFκB activation, inhibits TSC1 and thereby activates mTORC1 21,22. As mTORC1-mediated phosphorylation of lipin1 controls the promoter access of *sterol response element-binding protein 1* (SREBP-1) to target genes of lipid synthesis 23,24, tetracycline-mediated suppression of IKKβ/mTORC1 signalling may attenuate sebaceous lipogenesis 25. In contrast, addition of TNFα to SZ95 human sebocytes increases lipid droplet formation and upregulates both fatty acid synthase and SREBP-1 expression 26.

## Erythromycin and other macrolides

Erythromycin and other macrolides exert anti-inflammatory activity by attenuation of both TNFα-IKKβ and ERK1/2 signalling 27,28, thus increasing the inhibitory effect of TSC1/TSC2 towards Rheb.

## Isotretinoin and *all-trans*-retinoic acid

Circumstantial evidence supports the view that isotretinoin (*13-cis* retinoic acid) increases nuclear FoxO1 levels 29,30. Isotretinoin isomerizes to *all-trans* retinoic acid (ATRA), which interacts with retinoic acid receptors (RARs) 31. ATRA/RAR signalling induces secondary responses in gene expression involving FoxO proteins 32. ATRA increases the expression of FoxO3a in neuroblastoma cells 33. FoxO3a mediates ATRA-induced granulocytic differentiation and apoptosis in acute promyelocytic leukaemia (APL) cells 34. Notably, FoxO3a is a strong inducer of FoxO1 35. ATRA treatment for APL cells translocates FoxO3a into the nucleus 34. In this regard, the ATRA-induced protein *stimulated by retinoic acid 8* (STRA8), which physically interacts with CRM1, may modify CRM1-mediated nuclear FoxO1 export 36.

Isotretinoin induces cell cycle arrest in SEB-1 sebocytes with increased expression of the cell cycle inhibitor p21 37. Upregulation of the FoxO1 target gene p21 could thus explain isotretinoin-induced sebocyte apoptosis 38. Furthermore, isotretinoin/ATRA-induced FoxO upregulation may stimulate autophagy by FoxO-mediated suppression of mTORC1 2,39.

## Azelaic acid

Azelaic acid (AzA) dose-dependently suppresses mitochondrial respiration in rat liver by competitive inhibition of mitochondrial enzymes including succinic dehydrogenase decreasing ATP generation and increasing mitochondrial ROS release 40,41. Another mitochondrial toxin, 3-nitropropionic acid (3-NP), selectively inhibits mitochondrial succinic dehydrogenase 42,43. 3-NP treatment-induced mitochondrial ROS leakage induces apoptosis in neuronal cells 42,43. In mouse follicular granulosa cells, 3-NP increases ROS formation, raises nuclear FoxO1 levels and induces apoptosis 7. AzA-mediated mitochondrial ROS release may upregulate FoxO expression and inhibit mTORC1, thus mimicking ROS-stimulated effects of BPO treatment.

## Antiandrogens

Androgen-mediated stimulation of mTORC2 and consecutive mTORC2-mediated activation of Akt inhibit FoxO1 activity 44 and stimulate androgen receptor transcriptional activity 45. The therapeutic effectiveness of antiandrogens in female acne patients may thus be related to impaired androgen/TORC2/Akt-mediated suppression of TSC2 inhibition as well as reduced Akt-mediated nuclear FoxO1 export, thus attenuating mTORC1 activity.

## Conclusion

Translational evidence supports the hypothesis that antiacne agents may either attenuate mTORC1 activity indirectly by upregulating nuclear FoxO levels or by direct inhibition of mTORC1 (Fig. 1). This hypothesis may allow the rational development of new antiacne drugs such as FoxO enhancers or synthetic and natural mTORC1 inhibitors like resveratrol and epigallocatechin-3-gallate (Table S1).

## Supporting Information

**Data S1.** Full-length hypothesis.

**Figure S1.** FoxO shuttling between nucleus and cytoplasm.

**Figure S2.** Drug-mediated modifications of nuclear FoxO trafficking.

**Figure S3.** Inhibition of mTORC1 activity by antiacne agents.

**Table S1.** Action of commonly used and potential new antiacne drugs correcting imbalanced FoxO1-/mTORC1 signalling in acne.
