# Supplementary material for: Are therapeutic effects of antiacne agents mediated by activation of FoxO1 and inhibition of mTORC1?
Source: Exp Dermatol. 2013 Jun 25;22(7):502–4. doi: 10.1111/exd.12172 (PMC3746104; doi:10.1111/exd.12172)
Supplement: Supplementary file 1 [file exd0022-0502-SD1.doc]

**Supporting Information**

**FULL-LENGTH HYPOTHESIS**

**Are therapeutic effects of anti-acne agents mediated by activation of FoxO1 and inhibition of mTORC1?**

**Introduction**

Acne in Westernized countries is an epidemic skin disease during adolescence, and exhibits an increasing persistence into adult life (1-5). Acne has been identified as a disease of Western civilization (6), recently associated with increased body mass index and insulin resistance (7-11). Age-related diseases of civilization like obesity, diabetes, and cancer are associated with nutrient overload and anabolic states of metabolism mediated by exaggerated mTORC1 signaling (12-14). Thus, acne has been proposed to represent a visible, anabolic mTORC1-driven disease of the pilosebaceous follicle (15). Adolescence and young adulthood, a time of increased endocrine insulin/IGF-1 signaling (IIS), is often superimposed upon by enhanced IIS from a Western diet composed of high glycaemic load, high fat intake (abundant calorie intake) and increased steroid hormones in commercial milk as well as by increased branched chain-amino acid (BCAA)-mediated stimulation of mTORC1 released by milk and dairy proteins (16). Acne-promoting genetic mutations of *TNF*, *IGF1* or *AR with shorter CAG repeats* are all integrated at the regulatory level of mTORC1 and increase mTORC1 activity. In contrast, mTORC1 activity is suppressed by FoxO1 signaling (17). Evidence derived from translational research supports the concept that isotretinoin´s mode of action is associated with increased nuclear FoxO1 signaling (18, 19). Thus, the question arose whether anti-acne agents may directly attenuate mTORC1 signaling or may promote FoxO1-mediated suppression of mTORC1.

**Benzoyl peroxide**

The standard topical therapy of acne vulgaris is benzoyl peroxide (BPO), which is applied in high concentrations commonly ranging from 3% to 10% (20, 21). BPO is an organic compound of the peroxide family and penetrates into all skin layers and structures of the pilosebaceous follicle in a concentration-dependent fashion (22, 23).

BPO´s major mode of action is believed to reduce the growth of *Propionibacterium acnes.* However, BPO-mediated extinction of *P. acnes* takes only minutes, whereas clinical improvement of acne takes several weeks of continued BPO treatment. Thus, there appears to be another overlooked mode of action of BPO in the treatment of acne. This most likely involves reactive oxygen (ROS)-mediated activation of FoxO signaling. There is good reason to assume that BPO activates cellular oxidative stress-inducible kinases, Jun-N-terminus kinase (JNK) and STE20-like protein kinase 1 (MST1), which increase nuclear FoxO levels (24). Remarkably, the activation of FoxOs, initiated by JNK- and MST1-mediated FoxO phosphorylation is dominant to the inhibitory phosphorylation of FoxOs mediated by Akt (Fig. S1 and Fig. S2) (25). Thus, BPO/ROS/JNK/MST1-activated FoxO signaling may be the crucial effector mechanism of BPO treatment of acne, which counteracts IIS/Akt-mediated inactivation of FoxO transcription factors (Fig. S2) (15, 18).

Thus, BPO/ROS-triggered activation of the oxidative stress-inducible kinases may upregulate nuclear FoxO levels and appears to be the predominant pharmacologic mode of action of peroxide treatment of acne, which may counterbalance insulinotropic Western diet-induced nuclear FoxO deficiency (Fig. S1). Furthermore, BPO-mediated FoxO upregulation may attenuate downstream mTORC1 signaling. Thus, it should be expected that BPO treatment would reduce mTORC1-dependent growth and cell proliferation of keratinocytes and sebocytes. Notably, mTORC1 controls the G1/S transition and G2/M progression of the cell cycle (26).

It has been shown by Gloor *et al.* that BPO treatment decreases the size of golden hamster ear sebaceous glands (SGs) and reduces the number of sebocytes entering the S-phase of the cell cycle (27). Similar antiproliferative effects have been confirmed by autoradiographic studies of human SGs (28, 29). Although less efficient than ATRA, BPO decreases the size and number of corneocytes and reduces comedo formation in the rabbit ear microcomedo prevention assay (30, 31). In fact, recent evidence derived from follicular granulosa cells confirmed that the addition of hydrogen peroxide stimulates nuclear translocation of FoxO1 (32).

BPO/ROS-mediated nuclear FoxO1 upregulation may thus suppress mTORC1 activity, cell growth and cell proliferation. It is well known that activated FoxOs (FoxO1, FoxO3, and FoxO4) induce the expression of Sestrin3, which activates AMPK to inhibit mTORC1 in a TSC2-dependent manner (Fig. S3) (33). In addition, AMPK activation by increased ROS generation represents another mechanism inhibiting mTORC1. In response to increased cellular ROS levels, the cellular damage and cytoplasmic ROS sensor *ataxia-telangiectasia mutated* (ATM) activates TSC2 via the LKB1/AMPK metabolic pathway to repress mTORC1 and to induce autophagy (34). There is substantial evidence that not only endogenous ROS but also exogenous ROS, by direct addition of hydrogen peroxide to cells, potently and rapidly induces mTORC1 repression (35). It is thus not surprising that the clinical efficacy of a hydrogen peroxide cream was comparable to the anti-acne effects of BPO (36, 37).

Taken together, BPO most likely acts as a follicular ROS generator that upregulates nuclear FoxO1 levels and promotes FoxO/Sestrin3/AMPK/TSC2 and ROS/ATM/AMPK/TSC2-mediated mTORC1 suppression thereby inhibiting cell cycle progression resulting in reduced keratinocyte and sebocyte growth and cell proliferation (Fig. S3).

**Tetracyclines**

A mainstay of systemic treatment of moderate to severe inflammatory acne is doxycycline, the most commonly prescribed oral antibiotic in the treatment of acne (38). It has been known for a long time that doxycycline inhibits bacterial growth and exerts antiinflammatory effects even when administered in subantibacterial doses (39).

There is accumulating evidence that FoxO transcription factors are intimately involved in the regulation of inflammation, innate and adaptive immunity and expression of antimicrobial peptides (40-46). The transport of FoxO proteins through the nuclear pore is dependent on active-transport mechanisms (47, 48). Transport across the nuclear pore complex requires adaptor proteins that mediate either import or export. These adaptors are importin or exportin receptors, respectively (49). Importins and exportins recognize specific nuclear localization signals (NLSs) and nuclear export signals (NESs) present in the protein to be exchanged. Previous studies of FoxO1 have identified an NLS in the C-terminal basic region of the DNA-binding domain, and a leucine-rich, leptomycin-sensitive NES located further downstream of FoxO1. The evolutionary conserved protein *chromosomal region maintenance protein1* (CRM1, also known as exportin-1) recognizes several NESs including those of FoxO proteins (47, 50). Therefore, FoxO1 binding to CRM1 is a requirement for FoxO1´s nuclear export (Fig. S2) (50-52).

Intriguingly, recent evidence has been provided in a bitransgenic mouse lung tumor model that, in mice treated with doxycycline, CRM1 expression decreases dramatically in comparison to untreated mice (53). From this observation it can be suggested that doxycycline may exert its antiinflammatory action by decreasing CRM1 expression, a pivotal regulatory event that would enhance nuclear FoxO levels. As FoxO proteins exert antiinflammatory and immune modulatory effects, doxycycline-mediated nuclear FoxO1 retention may represent a new explanation for the antiinflammatory potential of tetracyclines in the treatment of acne and rosacea (Fig. S2).

Many signaling pathways that activate NF-κB converge at the level of the kinase IKKβ. Important stimuli leading to IKKβ activation include the inflammatory cytokines tumor necrosis factor-α (TNFα) and interleukin-1β (IL-1β)(54). There is accumulating evidence that doxycycline inhibits the secretion of TNFα and IL-1β in monocyte/macrophages and other cell systems (55, 56). IKKβ, the crucial kinase of proinflammatory NFκB activation, phosphorylates and thereby inhibits TSC1 (57, 58). The inhibition of TSC1 leads to activation of Rheb and finally of mTORC1 (57, 58). mTORC1 controls the promoter access of the key transcription factor of lipogenesis *sterol response element binding protein-1* (SREBP-1) to target genes regulating fatty acid, acyl glycerol and glycerophospholipid synthesis and the metabolism by mTORC1-mediated phosphorylation of lipin1 (59, 60). Tetracycline-mediated attenuation of IKKβ activity may thus partially repress IKKβ/TSC1-stimulated mTORC1 activation, which may not only exert antiinflammatory effects but may also attenuate mTORC1/lipin1/SREBP-1c-mediated sebaceous lipogenesis. Although only very limited former studies have investigated the effect of tetracycline treatment on sebaceous lipogenesis, the study of Beveridge *et al.* (61) reported a moderate decrease (-15.4%) of sebum excretion from 39.5 to 33.4 mg/10 cm^2^/3h in 5 of 6 acne patients treated with tetracylcline (2x 250 mg/day) for 8 weeks. In contrast, Choi *et al.* (62) have recently demonstrated that the addition of TNFα to SZ95 human sebocytes increases lipid droplet formation with upregulated expression of fatty acid synthase (FAS) and SREBP-1 through JNK- and PI3K/Akt pathways resulting in mTORC1 activation. Recent evidence underlines the importance of mTORC1 activity for SREBP-1 signaling (63).

Taken together, indirect evidence derived from translational research allows the suggestion that tetracyclines may exert their therapeutic anti-acne effects by nuclear retention of FoxO transcription factors as well as by attenuation of IKKβ/TSC1-mediated mTORC1 activation and mTORC1/lipin1-driven lipogenesis (Fig. S3).

**Erythromycin and other related macrolides**

Erythromycin is a commonly used topical anti-acne agent. This macrolide exerts antiinflammatory effects (64, 65). Recent evidence underlines that erythromycin and other macrolides like azithromycin, clindamycin, and clarithromycin exert antiinflammatory activity due to attenuation of TNFα-IKKβ- as well as of ERK1/2 signaling (66-71), thus increasing the inhibitory effect of TSC1/TSC2 towards Rheb. Macrolide-mediated attenuation of ERK1/2 signaling increases the inhibitory activity of TSC2 towards Rheb. Macrolide-mediated suppression of IKKβ enhances the inhibitory acitivity of TSC1 towards Rheb. Both pathways thus stabilize the inhibitory function of the TSC1/TSC2 complex, which apparently leads to an attenuation of mTORC1 activity and mTORC1-driven protein and lipid synthesis.

**Isotretinoin and *all-trans* retinoic acid**

Substantial indirect evidence has been provided that isotretinoin, the most potent antiinflammatory and sebum-suppressive drug in the treatment of acne, may increase nuclear levels of FoxO1 (19). Further support for the *isotretinoin-FoxO1-hypothesis* (19) comes from a recent genome-wide analysis of FoxO1 binding in mouse hepatic chromatin (72). It is well known that the phosphoenolpyruvate carboxykinase gene (*Pck1*) and the pyruvate dehydrogenase kinase isoenzyme 4 gene (*Pdk4*) are retinol and ATRA-responsive genes (73-76). At the promoter level these genes are not only regulated by retinoic acid response elements but also by FoxO1 (74, 77-79). *Pck1-* and *Pdk4* gene expression of retinol and ATRA-stimulated cells was significantly reduced when FoxO1 was deleted (72). Thus, maximal induction of *Pck1* and *Pdk4* appears to require the promoter activation of both the retinoic acid response elements as well as of FoxO1-binding sequences. This view is compatible with isotretinoin/ATRA/FoxO1-stimulated activation of *Pck1* and *Pdk4*, which appears to be reduced when FoxO1 is deleted. This mechanism explains increased gluconeogenesis with elevated glucose serum levels in patients systemically treated with higher doses of isotretinoin (15, 19).

Isotretinoin (*13-cis* retinoic acid) is regarded as a prodrug mediating its regulatory effects after isomerization to *all-trans* retinoic acid (ATRA), which interacts with retinoic acid receptors (80). Binding of ATRA initiates changes in interactions of retinoic acid receptors (RARs)/retinoid X receptor (RXRs) with corepressor and coactivator proteins, activating transcription of primary target genes. Importantly, ATRA/RAR signaling induces secondary responses in gene expression encoding transcription factors and signaling proteins that further augment a whole cascade of gene expression (81). FoxO proteins are transcription factors of the secondary response, which activates FoxO-dependent target genes to generate the whole spectrum of retinoid-mediated transcriptional regulation (81). ATRA increases the expression of FoxO3a in neuroblastoma cells (82). FoxO3a has also been identified as a key regulator for ATRA-induced granulocytic differentiation and apoptosis in acute promyelocytic leukemia (83). ATRA-inducible FoxO3a is a strong inducer of the transcription factor FoxO1 at the promoter level of FoxO1 (84). Remarkably, treatment of acute promyelocytic leukemia cells with ATRA reduced FoxO3a phosphorylation and translocated FoxO3a into the nucleus. Thus, it is conceivable that ATRA exerts its gene regulatory mode of action by interfering with FoxO nuclear transport mechanisms. In this regard it is most exciting that the ATRA-induced protein *stimulated by retinoic acid 8* (STRA8) physically interacts with CRM1, which may modify CRM1-mediated nuclear FoxO1 export (85) (Fig. S2).

Upregulation of FoxO1 transcription factors could explain the mechanism of isotretinoin-induced sebocyte apoptosis. Isotretinoin treatment induces cell cycle arrest in SEB-1 sebocytes and increases the expression of the cell cycle inhibitor p21 (86). Remarkably, p21 expression is upregulated at the promoter level by FoxO1 (79). Moreover, FoxOs induce apoptosis by regulating the expression of multiple pro- and anti-apoptotic proteins. Upregulation of the death receptor ligands FasL and TRAIL will induce apoptosis through activation of the extrinsic apoptotic pathway, while FoxO-regulated expression of Bim, Puma, BcLXI, and Pink1 can induce apoptosis via the mitochondria-dependent intrinsic pathway (79). Isotretinoin/ATRA-induced upregulation of FoxO1 and FoxO3 may not only induce apoptosis but may enhance autophagy by FoxO-mediated suppression of mTORC1 (17, 33). Thus, isotretinoin/ATRA-induced FoxO-mediated cell cycle arrest, apoptosis and FoxO/mTORC1-mediated autophagy may explain the histological changes in human skin biopsies that demonstrate a drastic decrease in the size, shape and lipid content of SGs after oral isotretinoin treatment (87).

Treatment of acne with isotretinoin and ATRA may also attenuate mTORC1 by suppressing peripheral androgen signaling. Isotretinoin-mediated upregulation of nuclear FoxO1 (18, 19), which is an androgen receptor (AR) cosuppressor, may attenuate AR transactivation and downstream androgen signaling (18, 19, 88-90).

The cytochrome P450 enzyme CYP3A4 is highly expressed in the liver but is also found in significant concentrations in the skin (91). CYP3A4 facilitates oxidative inactivation of androgens such as testosterone and androstanediol to its inactive 6β-OH metabolites (92-94). Remarkably, retinoids like *9-cis* retinoic acid (9-cis RA), *13-cis* RA (isotretinoin) and ATRA can effectively induce CYP3A4 gene expression at the promoter level through the RXR/constitutive androstane receptor (CAR) pathway (95). Supposing that retinoids induce CYP3A4 levels in the skin of acne patients, intracrine androgen metabolism may be suppressed. In this regard, increased FoxO1-mediated AR suppression and CYP3A4-mediated androgen (AR ligand) inactivation may impair androgen signaling. Reduced androgen-mediated stimulation of mTORC2 may ultimately result in diminished mTORC1 activity. This line of thought is supported by the pathophysiology of chloracne, a marker disease of systemic intoxication by 2,3,7,8-tetrachlorodibenzo-p-dioxin (TCDD) and related polycyclic aromatic hydrocarbons (PAHs), which exhibits decreased lipogenesis and SG involution as most prominent clinical signs of the disease (96). PAHs including TCDD activate CYP3A4 gene transcription through human pregnane X receptor (97). Increased androgen inactivation by over-expressed PAH-stimulated CYP3A4 expression thus offers a reasonable explanation for SG involution and suppressed sebaceous lipogenesis.

It is thus conceivable that combining a retinoid like adapalene with BPO, both apparently inducing independent pathways that increase nuclear FoxO retention, will demonstrate synergistic effects on inflammatory acne lesions (98).

**Azelaic acid**

Azelaic acid (AzA) is a naturally occurring 9-carbon dicarboxylic acid that exerts beneficial clinical effects in the treatment of inflammatory skin disorders like acne vulgaris, papulopustular rosacea and perioral dermatitis (99-101).

In isolated rat liver mitochondria, AzA inhibited mitochondrial respiration in a dose-dependent fashion. In particular, experiments on submitochondrial particles showed that AzA competitively inhibits the mitochondrial enzymes thioredoxin reductase, NADPH cytochrome P450 reductase, NADH dehydrogenase, succinic dehydrogenase and H_2_CoQ-Cytochrome C oxidoreductase resulting in decreased generation of ATP and release of ROS from mitochondria (102, 103).

Another natural mitochondrial toxin, 3-nitropropionic acid (3-NP) selectively inhibits the succinic dehydrogenase complex (complex II) in the mitochondrial electron transport chain, like AzA (104, 105). By inhibiting the respiratory chain and disturbing mitochondrial membrane integrity, 3-NP treatment induces ROS leakage from damaged mitochondria, reduces ATP production and induces apoptosis in neuronal cells (104, 105). In mouse follicular granulosa cells 3-NP treatment increases ROS generation and FoxO1 levels (32). In this cell system, 3-NP as well as H_2_O_2_-induced oxidative stress upregulates FoxO1 expression and promotes FoxO1-driven follicular graulosa cell apoptosis (32). Thus, in analogy to the anti-mitochondrial effects of 3-NP, AzA-mediated disturbance of mitochondrial integrity with resulting ROS release may upregulate FoxO expression, which via enhanced Sestrin3 expression and AMPK activation inhibits mTORC1. Furthermore, decreased cellular ATP levels and concomitant increases of AMP activate AMPK, which suppresses mTORC1 activation. AMPK phosphorylates TSC2 and Raptor, thereby suppressing mTORC1 activity (106-108). Thus, AzA-mediated attenuation of mitochondrial respiration, increasing ROS-mediated generation of FoxO1 and decreasing the cell´s energy supply might be major modes of action of AzA resulting in mTORC1 suppression (Fig. S3).

Moreover, AzA at 20 mM, a concentration achievable following topical application of a 15% gel, suppresses ultraviolet B light-induced IL-1β, IL-6 and TNFα mRNA expression and protein secretion (109). Thus, AzA-mediated suppression of TNFα/IKKβ signaling may represent a further mechanism which attenuates overactivated mTORC1 signaling in acne (57, 58) (Fig. S3).

In an experimental model of cellular senescence, AzA reduces the senescence-like phenotype in PUVA-irradiated human dermal fibroblasts and increases PPARγ transcriptional activity (110). However, it is unlikely that AzA-mediated upregulation of PPARγ plays a therapeutic role in sebocyte regulation in acne treatment as PPARγ is an important transcription factor co-stimulating sebaceous lipogenesis (111-114). Furthermore, it has been demonstrated that PPARγ activity is inhibited by FoxO1 but stimulated by mTORC1 (115-117).

**Antiandrogens**

Androgen signaling via the androgen receptor (AR) is of great importance for the generation of muscle mass and cellular amino acid (AA) influx. Androgen signaling promotes AA-stimulated activation of Rag/Ragulator-mediated activation of mTORC1. It has been demonstrated in prostate cancer cells, that androgen deprivation therapy downregulates the expression of L-type amino acid transporter-3 (LAT3), which is important to maintain intracellular leucine levels for mTORC1 activation (118). Inhibition of either LAT3 or LAT1 decreases growth of prostate cancer cells (118). Antiandrogen treatment of acne thus may attenuate AA-mediated activation of mTORC1 (Fig. S3).

Furthermore, androgen signaling has been demonstrated to inhibit FoxO1 and to stimulate mTORC1. In prostate cancer cells, androgens increase TORC2-mediated activation of Akt (119, 120). Androgen-mediated TORC2/Akt activation inhibits the activity of FoxO1 (120). Moreover, androgen-mediated TORC2-mediated Akt phosphorylation stimulates AR transcriptional activity (121).

Taken together, there exist close interactions between AR signaling, mTORC2-, Akt-, FoxO1-, and mTORC1 activity. The mode of action of antiandrogens in the treatment of acne may be related to impaired androgen/TORC2/Akt-mediated inhibition of FoxO1 and suppression of mTORC1 activity coordinating androgen-driven anabolic processes like SG hyperplasia and sebaceous lipid synthesis.

**Potential new anti-acne drugs enhancing nuclear FoxO1 activity**

**Vitamin D.** Sebocytes and keratinocytes are 1,25-dihydroxyvitamin D3 (1,25D3)-responsive target cells, express vitamin D receptor (VDR), and vitamin D-metabolizing enzymes (122-124). Recent experimental evidence indicates that vitamin D analogs may be effective in the treatment of acne (125, 126). In sebocytes, keratinocytes and other cells, 1,25D3 signals through the nuclear VDR and regulates growth and differentiation (125). Accumulating evidence indicates that 1,25D3 and FoxO proteins similarly regulate common target genes. In fact, ligand-bound VDR regulates the posttranslational modification and function of FoxO proteins (127). 1,25D3 treatment enhances binding of FoxO3a and FoxO4 to promoters of FoxO target genes and blocks growth factor-induced FoxO protein nuclear export. Furthermore, VDR associates directly with FoxO proteins and regulators, the sirtuin 1 (Sirt1) class III histone deacetylase (HDAC), and protein phosphatase 1. 1,25D3 treatment rapidly induces FoxO deacetylation and dephosphorylation, consistent with FoxO activation (127). In contrast, ablation of VDR expression enhances FoxO3a phosphorylation. 1,25D3-dependent cell cycle arrest is blocked in FoxO3a-deficient cells, indicating that FoxO proteins are key downstream mediators of the antiproliferative actions of 1,25D3. These observations link 1,25D3 signaling through VDR directly to FoxO function and provide the molecular basis for the synergistic antiproliferative and antiinflammatory signaling of vitamin D and FoxOs.

Surprisingly, no study investigated a possible correlation between the severity and grade of inflammation of acne and serum 1,25D3 levels in acne patients. However, comedolytic effects of topically applied active vitamin D3 analogs have been demonstrated on pseudocomedones in the rhino mouse (128, 129). Remarkably, oral isotretinoin treatment of acne patients significantly increases serum levels of 1,25D3 (130). Increased 1,25D3 serum vitamin D levels after isotretinoin treatment may thus represent another VDR-mediated mechanism that enhances nuclear FoxO levels. Furthermore, isotretinoin-mediated increase of serum 1,25D3 may explain the increased risk for skeletal hyperostoses after prolonged high-dose isotretinoin treatment.

**Leptomycin B.** The herbal fungicide leptomycin B (LMB) is a specific inhibitor of the nuclear export factor CRM1 (exportin 1) (131, 132). LMB specifically inhibits CRM1-FoxO1 interaction by binding to the LMB-sensitive NES motif of FoxO1 (133). Thus, FoxO proteins accumulate in the nucleus after treatment with LMB (134-136). The fact that LMB enhances nuclear FoxO accumulation suggests the prediction that topical treatment of acne with LMB might be a potential new anti-acne therapy. LMB has already been shown to exert antiinflammatory action in contact dermatitis and inhibited the expression of cyclooxygenase-2 (COX-2) and matrix metalloproteinases (MMP)-3 and MMP-9 (137-139). Notably, increased expressions of COX-2 and of MMP-9 have been observed in SGs of acne patients (140, 141). Remarkably, isotretinoin, which has been predicted to increase nuclear levels of FoxO1 (19), reduces the expression of COX-2 and MMP-9 (140-142).

The relation between LMB-mediated inhibition of CRM1, the nuclear FoxO exporter, with decreased expression of MMP-9 allows the assumption that LMB-induced inhibition of MMP-9 expression may be mediated by increasing nuclear FoxO levels. In fact, FoxO4 activates MMP-9 expression at the MMP-9 promoter (143). Isotretinoin, the prodrug of ATRA, reduces MMP-9 expression in a comparable fashion to LMB. This allows the speculation that the isotretinoin isomerization product ATRA may interfere with the regulation of the nuclear export machinery, most likely with CRM1 or other CMR1-interacting proteins like STRA8 or other retinoid-sensitive regulators of the nuclear core complex (80, 144).

**CRM1 inhibitors.** A chemical genetic screen has identified 42 compounds that inhibited FOXO1a nuclear export (145, 146). Of 42 positive compounds, 19 were identified as general CRM1 nuclear export inhibitors. Of the 19 small molecule inhibitors identified, 11 compounds were found to covalently modify CRM1 at cysteine 528 by a Michael-type reaction, similar to LMB (145). CRM1 inhibition, which increases nuclear FoxO levels, is a possible new pharmacologic strategy for the treatment of acne and other inflammatory skin diseases. The level of cargo binding to CRM1 appears to be dependent on the concentration of CRM1, which is rate limiting for nuclear export (147, 148). Downregulation of CRM1-expression by doxycycline thus represents a suitable mechanism to increase nuclear levels of FoxO1 (53). Other small molecular CRM1 inhibitors may be worth studying as new treatment options against acne.

**Metformin.** Acne of puberty, as well as all acne-associated syndromes, especially polycystic ovary syndrome (PCOS) is associated with insulin resistance (11, 149, 150). The “old” anti-diabetic drug metformin in combination with either spironolactone or simvastatin has been shown to improve acne in women with PCOS (151, 152). Treatment of PCOS with metformin improves overweight, insulin resistance and clinical signs of hyperandrogenism including acne (153). Notably, acne has been associated with increased body mass index (7-10). Metformin reduces body weight and activates AMPK resulting in mTORC1 inhibition (Fig. S3) (154, 155).

Recently, in glioma-initiating cells metformin has been identified as a therapeutic activator of FoxO3 (156). Metformin activated FoxO3 via AMPK stimulation and promoted differentiation of stem-like glioma-initiating cells into nontumorigenic cells (156). Thus, metformin via AMPK activation not only suppresses mTORC1 activation, but via AMPK activation increases nuclear FoxO3 levels (157). Remarkably, FoxO3 induces the expression of TSC1, the pivotal upstream suppressor of mTORC1 (158). Furthermore, FoxO3 induces the expression of Sestrin3, which inhibits mTORC1 by activation of AMPK (159). Moreover, FoxO3 at the promoter level upregulates the expression of FoxO1 (79). Thus, there is accumulating evidence that metformin exerts anti-proliferative and antiinflammatory activities by upregulation of FoxO3 and FoxO1.

**Potential new anti-acne drugs suppressing mTORC1 activity**

There is substantial evidence that acne is an anabolic skin disease of civilization driven by overactivated mTORC1 signaling (15). mTORC1 is regarded as the hub of the PI3K→Akt→mTORC1 pathway. PI3Ks and mTOR are related kinases, which share an evolutionarily related kinase domain, although the former is a lipid kinase and the latter is a protein kinase (160). As a result of their similar ATP sites, the prototypical PI3K inhibitors LY294002 and wortmannin inhibit both kinases, although the compounds have been primarily thought of as inhibitors of PI3Ks. Rapamycin has historically provided a means for selective mTOR inhibition, yet it is not a typical ATP competitive inhibitor (160). Several drugs and natural plant-derived polyphenols have been identified as mTOR kinase inhibitors that may have beneficial effects in the treatment of acne.

**Metformin**. The primary mode of metformin action is believed to operate via activation of the cell´s energy sensor AMPK (155, 156). AMPK phosphorylates TSC2 and Raptor, thereby suppresses mTORC1 activity (161, 162). It has recently been demonstrated that metformin treatment reduces airway inflammation and remodeling via activation of AMPK and reduces the activity of IKKβ (163). Reduced IKKβ activity has been demonstrated in white and red gastrocnemius muscles of obese rats after metformin treatment (164). Substantial evidence underlines that the TSC1-mTORC1 signaling pathway regulates inflammatory responses (165). Thus, by attenuating IKKβ-mediated TSC1 phosphorylation, metformin may reduce mTORC1 activity (Fig. S3).

Intriguingly, a further AMPK-independent mechanism of metformin-mediated mTORC1-inhibition has recently been identified. Metformin inhibits leucine-induced translocation of inactive mTORC1 to Rheb-enriched lysosomal membrane compartments and thereby reduces AA-induced mTORC1 activation (166). Thus, metformin counterbalances the adverse mTORC1-overactivating effects of Western diet mediated by high insulin/IGF-1 and leucine signaling (15).

**Natural plant-derived mTORC1 kinase inhibitors**

**Resveratrol** is a polyphenolic flavonoid from grapes and red wine with potential antiinflammatory, antioxidant, neuroprotective and anticancer properties that downregulates PI3K/Akt/mTORC1-signaling (167-171). Resveratrol has been shown to directly inhibit PI3K by targeting the class IA PI3K ATP-binding site in a competitive and reversible fashion (172). Resveratrol inhibits mTOR self-phosphorylation, the phosphorylation of mTORC1 targets S6K1 and 4E-BP1, and thus inhibits protein translation in hepatic cells (173). Thus, resveratrol acts as an indirect (via PI3K kinase) and direct (via mTOR kinase) inhibitor of mTORC1. These insights imply that resveratrol may exert beneficial therapeutic effects in the treatment of acne. In fact, topical treatment of facial acne vulgaris in 20 patients with a resveratrol-containing gel (0.01% weight/volume) significantly reduces the number of microcomedones, papules and pustules compared to vehicle control (174). Furthermore, resveratrol inhibits *P. acnes* growth and eradicates *P. acnes* biofilm formation (175, 176). These findings support a potential role of resveratrol in the future treatment of acne (177).

**Epigallocatechin-3-gallate** (EGCG), the major green tea catechin, is regarded as the active antiinflammatory and antiproliferative compound of green tea extracts (178-181). It has been demonstrated that topical 2% green tea lotion is effective in the treatment of mild-to-moderate acne vulgaris (182). After 6 weeks, the mean total lesion count and mean severity index of acne showed significant reductions of 58% and 39%, respectively (182). Furthermore, a 3% green tea emulsion significantly reduces sebum production in 10 healthy male volunteers after 8 weeks of treatment (183). Most recently, it has been demonstrated that topical application of EGCG to rabbit auricles reduced the size of the SGs (184). When applied to cultured human SZ95 sebocytes, EGCG strongly suppresses sebocyte mTORC1 activity, sebocyte proliferation and lipogenesis (184). Recently, EGCG has been shown to inhibit the AMPK/SREBP-1 pathway in SEB-1 sebocytes, and to inhibit *P. acnes* growth and to improve acne in an 8-week randomized split-face clinical trial with and without EGCG (185). EGCG-activated AMPK has been proposed to be the major mechanism of EGCG-mediated decrease of SREBP-1 (185). However, there is a further mechanism explaining the impact of EGCG on SREBP-1 regulation. AMPK by activating TSC2 inhibits mTORC1. mTORC1-mediated phosphorylation of lipin1 represents a most important regulatory step, which controls nuclear access of SREBP-1 to SREBP-1 target genes (59, 60, 63). Furthermore, EGCG in physiologically relevant concentrations has been shown to function directly as an ATP-competitive inhibitor of both PI3K and mTORC1 (186). Both resveratrol and EGCG are thus natural PI3K and mTORC1 lipid kinase inhibitors. In accordance, EGCG has been shown to activate AMPK, to suppress mTORC1 and 4E-BP1 and to inhibit lipogenesis in human hepatoma cells (187). Notably, the PI3K inhibitor EGCG upregulated nuclear FOXO levels in the worm *Caenorhabditis elegans* and attenuated the worm´s TOR signaling (188).

**Curcumin**, a natural polyphenol product isolated from the rhizome of the plant *Curcuma longa,* exerts antiproliferative effects and may present another class of clinically effective mTORC1 inhibitors (189). Curcumin attenuates mTORC1-mediated signaling pathways in cancer cells and inhibits phosphorylation of mTORC1 and its downstream targets, S6K1 and 4E-BP1 (190-192). Curcumin dissociates Raptor from mTORC1, resulting in inhibition of mTORC1 activity (193). Curcumin-loaded myristic acid microemulsions showed antimicrobial activity against *Staphylococcus epidermidis* and have been proposed as an alternative treatment of acne vulgaris (194).

**3,3´-Diindolylmethane (DIM)** is generated in the acidic environment of the stomach following dimerization of indole-3-carbinol monomers present in cruciferous vegetables. DIM suppresses signaling through Akt/mTORC1 pathways resulting in cell cycle arrest (195). Furthermore, DIM significantly inhibits both mTORC1 and Akt in PC3 PDGF-D cells, which is associated with decreased cell proliferation (196).

**Genistein,** a soy-derived isoflavone and phytoestrogen, has been shown to inhibit PI3K/Akt/mTORC1 signaling in mouse epidermal and human breast cancer cells (197).

**Caffeine**, a [xanthine](http://en.wikipedia.org/wiki/Xanthine) [alkaloid](http://en.wikipedia.org/wiki/Alkaloid), has been reported to inhibit PI3K kinase including mTORC1 (198, 199). Caffeine decreases the phosphorylation of the mTORC1 downstream targets S6K kinase, S6 ribosomal protein, 4E-BP1, thus elucidating caffeine´s mode of action as an inhibitor of mTORC1. Caffeine-induced autophagy has been shown to be mainly dependent on the PI3K/Akt/mTORC1 pathway (200).

**Silymarin,** a flavanolignan, extracted from the fruits and seeds of the milk thistle (*Silybum marianum L. Gaertn*.), has been shown to inhibit skin photocarcinogenesis and to exert antiinflammatory, antioxidant, and DNA repair mechanisms (201, 202). [Silymarin inhibits cell cycle progression and mTORC1 activity in activated human T cells (203) and thus represents another natural antiinflammatory polyphenol that inhibits mTORC1.](http://www.ncbi.nlm.nih.gov/pubmed/23121838)

Taken together, recent evidence indicates that various natural mTORC1-suppressing polyphenols either alone or in combination may be new promising agents for the treatment of acne.

**Rapamycin and rapalogs**

Future drug research and development in acne should focus on topical mTORC1 inhibitors. These may include the topical use of the immuno-suppressant allosteric mTORC1 inhibitor rapamycin and its analogs (termed rapalogs) like everolimus. Rapamycin and rapalogs first form a complex with the intracellular receptor FK506 binding protein 12 (FKBP12) and then bind a domain separated from the catalytic site of mTOR, blocking mTOR function (204).

**Synthetic mTOR kinase inhibitors**

Recently, a new generation of mTOR inhibitors, called mTORKinibs, which compete with ATP in the catalytic site of mTOR and inhibit both mTORC1 and mTORC2 with a high degree of selectivity, have been developed (204). These inhibitors bind to the ATP binding site of the kinase domain of mTOR and as a result inhibit the mTOR complexes, mTORC1 (rapamycin-sensitive) and mTORC2 (rapamycin-resistant) (205, 206).

**Vitamin D-mediated mTORC1 inhibition**

Vitamin D not only activates FoxO signaling but is also a strong inhibitor of mTORC1. It has recently been recognized that 1,25D3 stimulates the mRNA and protein expression of *DNA damage-inducible transcript 4* (DDIT4; also known as DNA damage response 1, REDD1) (207). DDIT4 knockdown by siRNA completely suppressed the antiproliferative effects of 1,25D3 (208). DDIT4 facilitates the assembly and activation of TSC1/TSC2 complex for eventual suppression of downstream mTORC1 activity (207). mTORC1 thus appears to be a master regulator target for the immunomodulatory and antiproliferative effects of vitamin D.

Vitamin D exerts further inhibitory effects on mTORC1 signaling. Vitamin D at the promoter level induces MAPK phosphatase-1 (MKP-1) (209-211), which is a most important feedback inhibitor of monocytes/macrophages during activated states of innate immune responses (212-214). Vitamin D/MKP-1-mediated attenuation of innate immunity may thus ameliorate TNFα signaling, which may lower mTORC1 activity via decreased TNFα/IKKβ/TSC1 signaling.

There exists another intriguing link between vitamin D signaling and androgen signaling. It has been demonstrated in prostate cells that vitamin D via VDR induces the expression of CYP3A4 and other genes that facilitate the oxidative inactivation of androgens such as testosterone and androstanediol (215). Supposing that vitamin D/CYP3A4-mediated reduction of intracrine androgens, which are involved in sebaceous lipogenesis, is an operative signaling mechanism in the human sebaceous gland, then vitamin D treatment of acne becomes a reasonable new therapeutic option. Vitamin D-mediated attenuation of androgen signaling may thus contribute to the antiproliferative mode of action due to attenuated androgen/mTORC2-Akt signaling.

**Conclusions**

The preceding viewpoint on the pathogenesis of acne in this journal suggests that epidemic acne of Western civilization results from deviant nutrient signaling with decreased nuclear FoxO1- and exaggerated mTORC1 activity (216). Provided that this conception is correct, then all anti-acne treatments should either increase FoxO1 activity or attenuate mTORC1 activity. In fact, the hypothesis presented supports the view that all remedies effective against acne may indeed either enhance FoxO1 and/or suppress mTORC1 activity (Table S1, Fig. S3). These insights thus open new avenues for a rational development of new drugs for the management of folliculo-occlusive disorders (Table S1). Of potential interest are synthetic small molecular weight mTORC1 inhibitors (TORKinibs) as well as natural plant-based mTORC1 inhibitors like resveratrol and epigallocatechin-3-gallate (EGCG) (217). Further potential new anti-acne drugs may be allosteric mTORC1 inhibitors like rapamycin and its analogs (rapalogs like everolimus). Indirect mTORC1 inhibitors like metformin, vitamin D and vitamin D analogs as well as CRM1-inhibitors like leptomycin B and its analogs, which enhance nuclear FoxO levels, may also be worth studying. Nevertheless, it is the strong believe of the authors that modern acne therapy should primarily correct the cause of epidemic acne, i.e., aberrant nutrient signaling induced by Western diet, by appropriate reduction of glycaemic load and milk/milk protein consumption (15). The majority of adolescents with moderate acne vulgaris may benefit from nutrition therapy (218, 219) and should only be supported by pharmacologic treatment, when dietary efforts have been demonstrated to be insufficient. Dietary and pharmacologic interventions in acne pursue a common objective: the attenuation of overstimulated mTORC1 signaling either induced by inappropriate diet and/or hereditable genetic polymorphisms (*IGF1*, *FGFR2*, *TNF*, *AR with reduced CAG repeats*), which converge in such over-stimulated mTORC1 signaling (216).

**References**

**1** James W D. Clinical practice. Acne. N Engl J Med 2005: **352:** 1463-1472.

**2** Collier C N, Harper J C, Carfadi J A *et al.* The prevalence of acne in adults 20 years and older. J Am Acad Dermatol 2008: **58:** 56-59.

**3** [Yentzer B A](http://www.ncbi.nlm.nih.gov/pubmed?term=Yentzer BA%5BAuthor%5D&cauthor=true&cauthor_uid=20919604), [Hick J](http://www.ncbi.nlm.nih.gov/pubmed?term=Hick J%5BAuthor%5D&cauthor=true&cauthor_uid=20919604), [Reese E L](http://www.ncbi.nlm.nih.gov/pubmed?term=Reese EL%5BAuthor%5D&cauthor=true&cauthor_uid=20919604) *et al.* Acne vulgaris in the United States: a descriptive epidemiology. Cutis 2010: **86:** 94-99.

**4** [Bhate K](http://www.ncbi.nlm.nih.gov/pubmed?term=Bhate K%5BAuthor%5D&cauthor=true&cauthor_uid=23210645), [Williams H C](http://www.ncbi.nlm.nih.gov/pubmed?term=Williams HC%5BAuthor%5D&cauthor=true&cauthor_uid=23210645). Epidemiology of acne vulgaris. Br J Dermatol 2013, **168:** 474-485.

**5** [Perkins A C](http://www.ncbi.nlm.nih.gov/pubmed?term=Perkins AC%5BAuthor%5D&cauthor=true&cauthor_uid=22171979), [Maglione J](http://www.ncbi.nlm.nih.gov/pubmed?term=Maglione J%5BAuthor%5D&cauthor=true&cauthor_uid=22171979), [Hillebrand G G](http://www.ncbi.nlm.nih.gov/pubmed?term=Hillebrand GG%5BAuthor%5D&cauthor=true&cauthor_uid=22171979) *et al.* Acne vulgaris in women: prevalence across the life span. J Womens Health (Larchmt) 2012: **21:** 223-230.

**6** Cordain L, Lindeberg S, Hurtado M *et al*. [Acne vulgaris: a disease of Western civilization.](http://www.ncbi.nlm.nih.gov/pubmed/12472346) Arch Dermatol 2002: **38:** 1584-1590.

**7** Di Landro A, Cazzaniga S, Parazzini F *et al.* Family history, body mass index, selected dietary factors, menstrual history, and risk of moderate to severe acne in adolescents and young adults. J Am Acad Dermatol 2012: **67:** 1129-1135.

**8** Halvorsen J A, Vleugels R A, Bjertness E *et al*. A population-based study of acne and body mass index in adolescents. Arch Dermatol 2012: **148:** 131-132.

**9** Tsai M C, Chen W C, Cheng Y W *et al*. Higher body mass index is a significant risk factor for acne formation in schoolchildren. Eur J Dermatol 2006: **16:** 251-253.

**10** Bourne S, Jacobs A. Observations on acne, seborrhoea, and obesity. Br Med J 1956; **1:** 1268-1270.

**11** Del Prete M, Mauriello M C, Faggiano A *et al*. Insulin resistance and acne: a new risk factor for men? Enodcrine 2012: **42:** 555-560.

**12** Hotamisligil G S, Erbay E. Nutrient sensing and inflammation in metabolic disease. Nature Rev Immunol 2008: **8:** 923-934.

**13** Dann S G, Selvaraj A, Thomas G. mTOR complex1-S6K1 signaling: at the crossroads of obesity, diabetes and cancer. Trends Mol Med 2007: **13:** 252-259.

**14** Zoncu R, Efeyan A, Sabatini D M. mTOR: from growth signal integration to cancer, diabetes and ageing. Nat Rev Mol Cell Biol 2011: **12:** 21-35.

**15** Melnik B. [Dietary intervention in acne: Attenuation of increased mTORC1 signaling promoted by Western diet.](http://www.ncbi.nlm.nih.gov/pubmed/22870349) Dermatoendocrinol 2012: **4:** 20-32.

**16** Melnik B C, John SM, Schmitz G. [Over-stimulation of insulin/IGF-1 signaling by Western diet may promote diseases of civilization: lessons learnt from Laron syndrome.](http://www.ncbi.nlm.nih.gov/pubmed/21699736) Nutr Metab (Lond). 2011: **8:** 41.

**17** Hay N. Interplay between FOXO, TOR, and Akt. Biochim Biophys Acta 2011: **1813:** 1965-1970.

**18** Melnik B C. [FoxO1 - the key for the pathogenesis and therapy of acne?](http://www.ncbi.nlm.nih.gov/pubmed/20151947) J Dtsch Dermatol Ges 2010: **8:** 105-114.

**19** Melnik B C. Isotretinoin and FoxO1: A scientific hypothesis. Dermatoendocrinol 2011: **3:** 141-165.

**20** [Williams H C](http://www.ncbi.nlm.nih.gov/pubmed?term=Williams HC%5BAuthor%5D&cauthor=true&cauthor_uid=21880356), [Dellavalle R P](http://www.ncbi.nlm.nih.gov/pubmed?term=Dellavalle RP%5BAuthor%5D&cauthor=true&cauthor_uid=21880356), [Garner S](http://www.ncbi.nlm.nih.gov/pubmed?term=Garner S%5BAuthor%5D&cauthor=true&cauthor_uid=21880356). Acne vulgaris. Lancet 2012: **379:** 361-372.

**21** [Sagransky M](http://www.ncbi.nlm.nih.gov/pubmed?term=Sagransky M%5BAuthor%5D&cauthor=true&cauthor_uid=19761357), [Yentzer B A](http://www.ncbi.nlm.nih.gov/pubmed?term=Yentzer BA%5BAuthor%5D&cauthor=true&cauthor_uid=19761357), [Feldman S R](http://www.ncbi.nlm.nih.gov/pubmed?term=Feldman SR%5BAuthor%5D&cauthor=true&cauthor_uid=19761357). Benzoyl peroxide: a review of its current use in the treatment of acne vulgaris. Expert Opin Pharmacother 2009: **10:** 2555-2562.

**22** [Nacht S](http://www.ncbi.nlm.nih.gov/pubmed?term=Nacht S%5BAuthor%5D&cauthor=true&cauthor_uid=7204686), [Yeung D](http://www.ncbi.nlm.nih.gov/pubmed?term=Yeung D%5BAuthor%5D&cauthor=true&cauthor_uid=7204686), [Beasley J N Jr](http://www.ncbi.nlm.nih.gov/pubmed?term=Beasley JN Jr%5BAuthor%5D&cauthor=true&cauthor_uid=7204686) *et al*. Benzoyl peroxide: percutaneous penetration and metabolic disposition J Am Acad Dermatol 1981: **4:** 31-37.

**23** [Yeung D](http://www.ncbi.nlm.nih.gov/pubmed?term=Yeung D%5BAuthor%5D&cauthor=true&cauthor_uid=6643790), [Nacht S](http://www.ncbi.nlm.nih.gov/pubmed?term=Nacht S%5BAuthor%5D&cauthor=true&cauthor_uid=6643790), [Bucks D](http://www.ncbi.nlm.nih.gov/pubmed?term=Bucks D%5BAuthor%5D&cauthor=true&cauthor_uid=6643790) *et al.* Benzoyl peroxide: percutaneous penetration and metabolic disposition. II. Effect of concentration. J Am Acad Dermatol 1983: **9:** 920-924.

**24** Huang H, Tindall D J. Dynamic FoxO transcription factors. J Cell Sci

2007:**120:** 2479-2487.

**25** Greer E L, Brunet A. FOXO transcription factors in aging and cancer. Acta Physiol (Oxf) 2008: **192:** 19-28.

**26** Wang X, Proud C G. Nutrient control of TORC1, a cell-cycle regulator. Cell 2009: **19:** 260-267.

**27** Gloor M, Klump H, Wirth H. Cytokinetic studies on the sebosuppressive effect of drugs using the example of benzoyl peroxide. Arch Dermatol Res 1980: **167:** 97-99.

**28** Fanta D. Klinische und experimentelle Untersuchungen über die Wirkung von Benzoylperoxid in der Behandlung der Akne. Hautarzt 1978: **29:** 481-486.

**29** Wirth H, Spürgel D, Gloor M. Untersuchungen zur Wirkung von Benzoylperoxid auf die Talgdrüsensekretion. Dermatol Monatsschr 1983: **169:** 289-293.

**30** Puschmann M. Klinisch-experimentelle Untersuchungen zum Wirkungsmechanismus von Benzoylperoxid. Hautarzt 1982: **33:** 257-265.

**31** Mezick J A, Thorne E G, Bhatia M C *et al.* (1987) The rabbit ear microcomedo prevention assay. A new model to evaluate antiacne agents. In: Models in Dermatology (Maibach H I, Lowe N J, eds) vol. 3. Karger: Basel, München, Paris, London, New York, New Delhi, Singapore, Tokyo, Sydney, 68-73.

**32** Shen M, Lin F, Zhang J *et al*. Involvement of the up-regulated FoxO1 expression in follicular granulosa cell apoptosis induced by oxidative stress. J Biol Chem 2012: **287:** 25727-25740.

**33** Chen C C, Jeon S M, Bhaskar P T *et al.* FoxOs inhibit mTORC1 and activate Akt by inducing the expression of Sestrin3 and Rictor. Dev Cell 2010: **18:** 592-604.

**34** Alexander A, Cai S L, Kim J *et al.* ATM signals to TSC2 in the cytoplasm to regulate mTORC1 in response to ROS. Proc Natl Acad Sci USA 2010: **107:** 4153-4158.

**35** Alexander A, Kim J, Walker C L. ATM engages the TSC2/mTORC1 signaling node to regulate autophagy. Autophagy 2010: **6:** 672-673.

**36** [Milani M](http://www.ncbi.nlm.nih.gov/pubmed?term=Milani M%5BAuthor%5D&cauthor=true&cauthor_uid=12740158), [Bigardi A](http://www.ncbi.nlm.nih.gov/pubmed?term=Bigardi A%5BAuthor%5D&cauthor=true&cauthor_uid=12740158), [Zavattarelli M](http://www.ncbi.nlm.nih.gov/pubmed?term=Zavattarelli M%5BAuthor%5D&cauthor=true&cauthor_uid=12740158). Efficacy and safety of stabilised hydrogen peroxide cream (Crystacide) in mild-to-moderate acne vulgaris: a randomised, controlled trial versus benzoyl peroxide gel. Curr Med Res Opin 2003: **19:** 135-138.

**37** [Capizzi R](http://www.ncbi.nlm.nih.gov/pubmed?term=Capizzi R%5BAuthor%5D&cauthor=true&cauthor_uid=15327558), [Landi F](http://www.ncbi.nlm.nih.gov/pubmed?term=Landi F%5BAuthor%5D&cauthor=true&cauthor_uid=15327558), [Milani M](http://www.ncbi.nlm.nih.gov/pubmed?term=Milani M%5BAuthor%5D&cauthor=true&cauthor_uid=15327558) *et al*. Skin tolerability and efficacy of combination therapy with hydrogen peroxide stabilized cream and adapalene gel in comparison with benzoyl peroxide cream and adapalene gel in common acne. A randomized, investigator-masked, controlled trial. Br J Dermatol 2004: **151:** 481-484.

**38** [Kircik L H](http://www.ncbi.nlm.nih.gov/pubmed?term=Kircik LH%5BAuthor%5D&cauthor=true&cauthor_uid=21061764). Doxycycline and minocycline for the management of acne: a review of efficacy and safety with emphasis on clinical implications. J Drugs Dermatol 2010: **9:** 1407-1411.

**39** [McKeage K](http://www.ncbi.nlm.nih.gov/pubmed?term=McKeage K%5BAuthor%5D&cauthor=true&cauthor_uid=20369903), [Deeks E D](http://www.ncbi.nlm.nih.gov/pubmed?term=Deeks ED%5BAuthor%5D&cauthor=true&cauthor_uid=20369903). Doxycycline 40 mg capsules (30 mg immediate-release/10 mg delayed-release beads): anti-inflammatory dose in rosacea. Am J Clin Dermatol 2010: **11:** 217-222.

**40** Peng S L. Forkhead transcription factors in chronic inflammation. Int J Biochem Cell Biol 2009: **42:** 482-485.

**41** Ouyang W, Beckett O, Flavell R A *et al.* An essential role of the Forkhead-box transcription factor Foxo1 in control of T cell homeostasis and tolerance. Immunity 2009: **30:** 358-371.

**42** Jeremy A H, Holland D B, Roberts S G *et al.* Inflammatory events are involved in acne lesion initiation. J Invest Dermatol 2003: **121:** 20-27.

**43** Dejean A S, Hedrick S M, Kerdiles Y M. Highly specialized role of Foxo transcription factors in the immune system. Antioxid Redox Signal 2011: **14:** 663-674.

**44** Kerdiles Y M, Beisner D R, Tinoco R *et al.* Foxo1 links homing and survival of naive T cells by regulating L-selectin, CCR7, and interleukin 7 receptor. Nat Immunol 2009: **10:** 176-184.

**45** Becker T, [Loch G](http://www.ncbi.nlm.nih.gov/pubmed?term="Loch G"%5BAuthor%5D), [Beyer M](http://www.ncbi.nlm.nih.gov/pubmed?term="Beyer M"%5BAuthor%5D) *et al.* FOXO-dependent regulation of innate immune homeostasis. [Nature](javascript:AL_get(this, 'jour', 'Nature.');) 2010: **463:** 369-373.

**46** Boehm A M, Khalturin K, Anton-Erxleben F *et al*. FoxO is a critical regulator of stem cell maintenance in immortal Hydra. Proc Natl Acad Sci USA 2012: **109:** 19697-19702.

**47** Van der Heide L P, Hoekman M F, Smid M P. The ins and outs of FoxO

shuttling: mechanisms of FoxO translocation and transcriptional regulation.

Biochem J 2004: **380:** 297-309.

**48** Mao L, Yang Y. Targeting the nuclear transport machinery by rational drug design. Curr Pharm Des 2013: **19:** 2318-2325.

**49** Komeili A, O’Shea E K. New perspectives on nuclear transport. Annu Rev Genet 2001: **35:** 341-364.

**50** [Vogt P K](http://www.ncbi.nlm.nih.gov/pubmed?term=Vogt PK%5BAuthor%5D&cauthor=true&cauthor_uid=15917664), [Jiang H](http://www.ncbi.nlm.nih.gov/pubmed?term=Jiang H%5BAuthor%5D&cauthor=true&cauthor_uid=15917664), [Aoki M](http://www.ncbi.nlm.nih.gov/pubmed?term=Aoki M%5BAuthor%5D&cauthor=true&cauthor_uid=15917664). Triple layer control: phosphorylation, acetylation and ubiquitination of FOXO proteins. Cell Cycle 2005: **4:** 908-913.

**51** Jacobs F M, van der Heide L P, Wijchers P J *et al*. Foxo6, a novel member of the FOXO class of transcription factors with distinct shuttling dynamics. J Biol Chem 2003: **278:** 35959-35967.

**52** Brownawell A M, Kops G J, Macara I G *et al.* Inhibition of nuclear import by protein kinase B (Akt) regulates the subcellular distribution and activity of the forkhead transcription factor AFX. Mol Cell Biol 2001: **21:** 3534-3546.

**53** Chen L, Moore J E, Samathanam C *et al.* CRM1-depedent p53 nuclear accumulation in lung lesions of a bitransgenic mouse lung tumor model. Oncol Rep 2011: **26:** 223-228.

**54** [Schmid J A](http://www.ncbi.nlm.nih.gov/pubmed?term=Schmid JA%5BAuthor%5D&cauthor=true&cauthor_uid=18308615), [Birbach A](http://www.ncbi.nlm.nih.gov/pubmed?term=Birbach A%5BAuthor%5D&cauthor=true&cauthor_uid=18308615). IkappaB kinase beta (IKKbeta/IKK2/IKBKB) - a key molecule in signaling to the transcription factor NF-kappaB. Cytokine Growth Factor Rev 2008: **19:** 157-165.

**55** [Cazalis J](http://www.ncbi.nlm.nih.gov/pubmed?term=Cazalis J%5BAuthor%5D&cauthor=true&cauthor_uid=18771379), [Bodet C](http://www.ncbi.nlm.nih.gov/pubmed?term=Bodet C%5BAuthor%5D&cauthor=true&cauthor_uid=18771379), [Gagnon G](http://www.ncbi.nlm.nih.gov/pubmed?term=Gagnon G%5BAuthor%5D&cauthor=true&cauthor_uid=18771379) *et al.* Doxycycline reduces lipopolysaccharide-induced inflammatory mediator secretion in macrophage and ex vivo human whole blood models. J Periodontol 2008: **79:** 1762-1768.

**56** [Jantzie L L](http://www.ncbi.nlm.nih.gov/pubmed?term=Jantzie LL%5BAuthor%5D&cauthor=true&cauthor_uid=20040243), [Todd K G](http://www.ncbi.nlm.nih.gov/pubmed?term=Todd KG%5BAuthor%5D&cauthor=true&cauthor_uid=20040243). Doxycycline inhibits proinflammatory cytokines but not acute cerebral cytogenesis after hypoxia-ischemia in neonatal rats. J Psychiatry Neurosci 2010: **35:** 20-32.

**57** Lee D F, Kuo H P, Chen C T *et al.* [IKK beta suppression of TSC1 links inflammation and tumor angiogenesis via the mTOR pathway.](http://www.ncbi.nlm.nih.gov/pubmed/17693255) Cell 2007: **130:** 440-455.

**58** Dan H C, Cooper M J, Cogswell P C *et al*. [Akt-dependent regulation of NF-{kappa}B is controlled by mTOR and Raptor in association with IKK.](http://www.ncbi.nlm.nih.gov/pubmed/18519641) Genes Dev 2008: **22:** 1490-1500.

**59** Porstmann T, Santos C R, Lewis C *et al.* A new player in the orchestra of cell growth: SREBP activity is regulated by mTORC1 and contributes to the regulation of cell and organ size. Biochem Soc Trans 2009: **37:** 278-283.

**60** Peterson T R, Sengupta S S, Harris T E *et al.* mTOR complex 1 regulates lipin1 localization to control the SREBP pathway. Cell 2011: **146:** 408-420.

**61** [Beveridge G W](http://www.ncbi.nlm.nih.gov/pubmed?term=Beveridge GW%5BAuthor%5D&cauthor=true&cauthor_uid=4240215), [Powell E W](http://www.ncbi.nlm.nih.gov/pubmed?term=Powell EW%5BAuthor%5D&cauthor=true&cauthor_uid=4240215). Sebum changes in acne vulgaris treated with tetracycline. Br J Dermatol 1969: **81:** 525-527.

**62** Choi J J, Park M Y, Lee H J *et al.* TNF-α increases lipogenesis via JNK and PI3K/Akt pathways in SZ95 human sebocytes. J Dermatol Sci 2012: **65:** 179-188.

**63** Bakan I, Laplante M. Connecting mTORC1 signaling to SREBP-1 activation. Curr Opin Lipidol 2012: **23:** 226-234.

**64** Tan A W, Tan H H. [Acne vulgaris: a review of antibiotic therapy.](http://www.ncbi.nlm.nih.gov/pubmed/15794732) Expert Opin Pharmacother 2005: **6:** 409-418.

**65** [Jain A](http://www.ncbi.nlm.nih.gov/pubmed?term=Jain A%5BAuthor%5D&cauthor=true&cauthor_uid=12546757), [Sangal L](http://www.ncbi.nlm.nih.gov/pubmed?term=Sangal L%5BAuthor%5D&cauthor=true&cauthor_uid=12546757), [Basal E](http://www.ncbi.nlm.nih.gov/pubmed?term=Basal E%5BAuthor%5D&cauthor=true&cauthor_uid=12546757) *et al.* Anti-inflammatory effects of erythromycin and tetracycline on Propionibacterium acnes induced production of chemotactic factors and reactive oxygen species by human neutrophils. Dermatol Online J 2002: **8:** 2.

**66** Kanoh S, Rubin B K. Mechanisms of action and clinical application of macrolides as immunomodulatory medications. Clin Microbiol Rev 2010: **23:** 590-615.

**67** Cosgrove B D, Alexopoulos L G, Hang T *et al*. Cytokine-associated drug toxicity in human hepatocytes is associated with signaling network dysregulation. Mol Biosyst 2010: **6:** 1195-1206.

**68** Desaki M, Okazaki H, Sunazuka T *et al.* Molecular mechanisms of anti-inflammatory action of erythromycin in human bronchial epithelial cells: possible role in the signaling pathway that regulates nuclear factor-kappaB activation. Antimicrob Agents Chemother 2004, **48:** 1581-1585.

**69** Wu L, Zhang W, Tian L *et al.* Immunomodulatory effects of erythromycin and its derivatives on human T-lymphocyte in vitro. Immunopharmacol Immunotoxicol 2007**: 29:** 587-596.

**70** Wu L, Lin J H, Bao K *et al*. In vitro effects of erythromycin on RANKL and nuclear factor-kappa B by human TNF-alpha stimulated Jurkat cells. Int Immunopharmacol 2009: **9:** 1105-1109.

**71** Ikegaya S, Inai K, Iwasaki H *et al*. Azithromycin reduces tumor necrosis factor-alpha production in lipopolysaccharide-stimulated THP-1 monocytic cells by modification of stress response and p38 MAPK pathway. J Chemther 2009: **21:** 396-402.

**72** Shin D J, Joshi P, Hong S H *et al.* Genome-wide analysis of FoxO1 binding in hepatic chromatin: Potential involvement of FoxO1 in linking retinoid signaling to hepatic gluconeogenesis. Nucleic Acids Res 2012: **2012:** 1-11.

**73** Licas P C, O´Brien R M, Mitchell J A *et al.* A retinoic acid response element is part of a pleiotropic domain in the phosphoenolpyruvate carboxykinase gene. Proc Natl Acad Sci USA 1991: **88:** 2184-2188.

**74** Shin D J, McGrane M M. Vitamin A regulates genes involved in hepatic gluconeogenesis in mice: phosphoenolpyruvate carboxykinase, fructose-1,6-biphosphatase and 6-phosphofructo-2-kinase/fructose-2,6-biphosphatase. J Nutr 1997: **127:** 1274-1278.

**75** Zhang Y, Li R, Chen W *et al.* Retinoids induced Pck1 expression and attenuated insulin-mediated suppression of its expression via activation of retinoic acid receptor in primary rat hepatocytes. Mol Cell Biochem 2011: **355:** 1-8.

**76** Scott D K, Mitchell J A, Granner D K. Identification and characterization of the second retinoic acid response element in the phosphoenolpyruvate carboxykinase gene promoter. J Biol Chem 1996: **271:** 6260-6264.

**77** Kang H W, Bhimidi G R, Odom D P *et al.* Altered lipid catabolism in the vitamin A deficient liver. Mol Cell Endocrinol 2007: **271:** 18-27.

**78** Cheng Z, White M F. Targeting forkhead box O1 from the concept to metabolic diseases: lessons from mouse models. Antiox Redox Signal 2011: **14:** 649-661.

**79** van der Vos K E, Coffer P J. The extending network of FOXO transcriptional target genes. Antioxid Redox Signal 2011: **14:** 579-592.

**80** Tsukada M, Schröder M, Roos T C *et al.* 13-cis retinoic acid exerts its specific activity on human sebocytes through selective intracellular isomerization to all-trans retinoic acid and binding to retinoid acid receptors. J Invest Dermatol 2000: **115:** 321-327.

**81** Gudas L J, Wagner J A. Retinoids regulate stem cell differentiation.

J Cell Physiol 2010: **226:** 322-330.

**82** Kim M J, Ahn K, Park S H *et al.* SIRT1 regulates tyrosine hydroxylase expression and differentiation of neuroblastoma cells via FOXO3a. FEBS Lett 2009: **583:** 1183-1188.

**83** Sakoe Y, Sakoe K, Kirito K *et al.* FOXO3A as a key molecule for all-trans retinoic acid-induced granulocytic differentiation and apoptosis in acute promyelocytic leukemia. Blood 2010: **115:** 3787-3795.

**84** Essaghir A, Dif N, Marbehant C Y *et al.* The transcription of FOXO genes is stimulated by FOXO3 and repressed by growth factors. J Biol Chem 2009: **284:** 10334-10342.

**85** Tedesco M, La Sala G, Barbagallo F *et al.* STRA8 shuttles bewteen nucleus and cytoplasm and displays transcriptional activity. J Biol Chem 2009: **284:** 35781-35793.

**86** Nelson A M, Gilliland K L, Cong Z *et al.* 13-cis retinoic acid induces apoptosis and cell cycle arrest in human SEB-1 sebocytes. J Invest Dermatol 2006: **126:** 2178-2189.

**87** Goldstein J A, Socha-Szott A, Thomsen R J *et al.* Comparative effect of isotretinoin and etretinate on acne and sebaceous gland secretion. J Am Acad Dermatol 1982: **6:** 760-765.

**88** Heemers H V, Tindall D J. Androgen receptor (AR) coregulators: a diversit

of functions converging on and regulating the AR transcriptional complex.

Endocr Rev 2007: **28:** 778-808.

**89** Yanase T, Fan W. Modification of androgen receptor function by IGF-1

signaling: implications in the mechanism of refractory prostate carcinoma. Vitam

Horm 2009: **80:** 651-666.

**90** Ma Q, Fu W, Li P *et al.* FoxO1 mediates PTEN suppression of androgen

receptor N- and C-terminal interactions and coactivator recruitment. Mol

Endocrinol 2009: **23:** 213-225.

**91** Yengi L G, Xiang Q, Pan J *et al.* Quantitation of cytochrome P450 mRNA

levels in human skin. Anal Biochem 2003: **316:**103-110.

**92** Gupta R P, Hollis B W, Patel S B *et al.* CYP3A4 is a human microsomal

vitamin D 25-hydoxylase. J Bone Miner Res 2004: **19:** 680-688.

**93** Di Marco A, Marcucci I, Verdirame M *et al.* Development and validation of a

high-throughput radiometric CYP3A4/5 inhibition assay using tritiated

testosterone. Drug Metab Dispos 2005: **33:** 349-358.

**94** Lee S J, Goldstein J A. Comparison of CYP3A4 and CYP3A5: the effects of

cytochrome b5 and NADPH-cytochrome P450 reductase on testosterone

hydroxylation activities. Drug Metab Pharmacokinet 2012: **27:** 663-667.

**95** Chen S, Wang K, Wan YJ. [Retinoids activate RXR/CAR-mediated](http://www.ncbi.nlm.nih.gov/pubmed/19686701)

[pathway and induce CYP3A.](http://www.ncbi.nlm.nih.gov/pubmed/19686701) Biochem Pharmacol 2010: **79:** 270-276.

**96** Ju Q, Fimmel S, Hinz N *et al.* 2,3,7,8-Tetrachlorodibenzo-p-dioxin alters

sebaceous gland cell differentiation in vitro. Exp Dermatol 2011: **20:** 320- 325.

**97** Kumagai T, Suzuki H, Sasaki T *et al.* Polycyclic aromatic hydrocarbons activate CYP3A4 gene transcription through human pregnane X receptor. Drug Metab Pharmacokinet 2012: **27:** 200-206.

**98** Zuliani T, Khammari A, Chaussy H *et al.* Ex vivo demonstration of synergistic effect of adapalene and benzoyl peroxide on inflammatory acne lesions. Exp Dermatol 2011: **20:** 850-853.

**99** [Del Rosso J Q](http://www.ncbi.nlm.nih.gov/pubmed?term=Del Rosso JQ%5BAuthor%5D&cauthor=true&cauthor_uid=16566285). The use of topical azelaic acid for common skin disorders other than inflammatory rosacea. Cutis 2006: **77:** 22-24.

**100** [Fleischer A B Jr](http://www.ncbi.nlm.nih.gov/pubmed?term=Fleischer AB Jr%5BAuthor%5D&cauthor=true&cauthor_uid=16566281). The evolution of azelaic acid. Cutis 2006: **77:** 4-6.

**101** [Jansen T](http://www.ncbi.nlm.nih.gov/pubmed?term=Jansen T%5BAuthor%5D&cauthor=true&cauthor_uid=19804495), [Melnik B C](http://www.ncbi.nlm.nih.gov/pubmed?term=Melnik BC%5BAuthor%5D&cauthor=true&cauthor_uid=19804495), [Schadendorf D](http://www.ncbi.nlm.nih.gov/pubmed?term=Schadendorf D%5BAuthor%5D&cauthor=true&cauthor_uid=19804495). Steroid-induced periorificial dermatitis in children - clinical features and response to azelaic acid. Pediatr Dermatol 2010: **27:** 137-142.

**102** [Passi S](http://www.ncbi.nlm.nih.gov/pubmed?term=Passi S%5BAuthor%5D&cauthor=true&cauthor_uid=6704136), [Picardo M](http://www.ncbi.nlm.nih.gov/pubmed?term=Picardo M%5BAuthor%5D&cauthor=true&cauthor_uid=6704136), [Nazzaro-Porro M](http://www.ncbi.nlm.nih.gov/pubmed?term=Nazzaro-Porro M%5BAuthor%5D&cauthor=true&cauthor_uid=6704136) *et al.* Antimitochondrial effect of saturated medium chain length (C8-C13) dicarboxylic acids. Biochem Pharmacol 1984: **33:** 103-108.

**103** [Passi S](http://www.ncbi.nlm.nih.gov/pubmed?term=Passi S%5BAuthor%5D&cauthor=true&cauthor_uid=2505463), [Picardo M](http://www.ncbi.nlm.nih.gov/pubmed?term=Picardo M%5BAuthor%5D&cauthor=true&cauthor_uid=2505463), [Mingrone G](http://www.ncbi.nlm.nih.gov/pubmed?term=Mingrone G%5BAuthor%5D&cauthor=true&cauthor_uid=2505463) *et al.* Azelaic acid - biochemistry and metabolism. Acta Derm Venereol Suppl (Stockh) 1989: **143:** 8-13.

**104** Kim G W, Copin J-C, Kawase M *et al.* Excitotoxicity is required for induction of oxidative stress and apoptosis in mouse striatum by the mitochondrial toxin, 3-nitropropionic acid. J Cereb Blood Flow Metab 2000: **20:** 119-129.

**105** Kim G W, Chan P H. Involvement of superoxide in excitotoxicity and DNA fragmentation in striatal vulnerability in mice after treatment with the mitochondrial toxin, 3-nitropropionic acid. J Cereb Blood Flow Metab 2002: **22:** 798-809.

**106** Inoki K, Zhu T, Guan K L. TSC2 mediates cellular energy response to control cell growth and survival. Cell 2003: **115:** 577-590.

**107** Shaw R J. LKB1 and AMPK control of mTOR signalling and growth. Acta Physiol (Oxf) 2009: **196:** 65-80.

**108** Gwinn D M, Shackelford D B, Egan D F *et al.* AMPK phosphorylation of raptor mediates a metabolic checkpoint. Mol Cell 2008: **30:** 214-226.

**109** [Mastrofrancesco A](http://www.ncbi.nlm.nih.gov/pubmed?term=Mastrofrancesco A%5BAuthor%5D&cauthor=true&cauthor_uid=20545756), [Ottaviani M](http://www.ncbi.nlm.nih.gov/pubmed?term=Ottaviani M%5BAuthor%5D&cauthor=true&cauthor_uid=20545756), [Aspite N](http://www.ncbi.nlm.nih.gov/pubmed?term=Aspite N%5BAuthor%5D&cauthor=true&cauthor_uid=20545756) *et al.* Azelaic acid modulates the inflammatory response in normal human keratinocytes through PPARgamma activation. Exp Dermatol 2010: **19:** 813-820.

**110** Briganti S, Flori E, Mastrofrancesco A *et al*. Azelaic acid reduced senescence-like phenotype in photo-irradiated human dermal fibroblasts: possible implication of PPARγ. Exp Dermatol 2013: **22:** 41-47.

**111** Downie M M, Sanders D A, Maier L M *et al.* Peroxisome proliferator-activated receptor and farnesoid X receptor ligands differentially regulate sebaceous differentiation in human sebaceous gland organ cultures in vitro. Br J Dermatol 2004: **151:** 766-775.

**112** Trivedi N R, Cong Z, Nelson A M *et al*. Peroxisome proliferator-activated recpetors increase human sebum production. J Invest Dermatol 2006: **126:** 2002-2009.

**113** Schuster M, Zouboulis C C, Ochsendorf F *et al.* Peroxisome proliferator-activated receptor activators protect sebocytes from apoptosis: a new treatment modality for acne? Br J Dermatol 2011: **164:** 182-186.

**114** Sertznig P, Reichrath J. Peroxisome proliferator-activated receptors (PPARs) in dermatology: challenge and promise. Dermatoendocrinol 2011: **3:** 130-135.

**115** Kim J J, Li P, Huntley J *et al*. FoxO1 haploinsufficiency protects against high-fat diet-induced insulin resistance with enhanced peroxisome-activator receptor gamma activation in adipose tissue. Diabetes 2009: **58:** 1275-1282.

**116** Kim J E, Chen J. Regulation of peroxisome proliferator-activated receptor-γ activity by mammalian target of rapamycin and amino acids in adipogenesis. Diabetes 2004: **53:** 2748-2756.

**117** Blanchard P G, Festuccia W T, Houde V P *et al*. Major involvement of mTOR in the PPARγ-induced stimulation of adipose tissue lipid uptake and fat accretion. J Lipid Res 2012: **53:** 1117-1125.

**118** Wang Q, Bailey C G, Ng C *et al*. Androgen receptor and nutrient signaling pathways coordinate the demand for increased amino acid transport during prostate cancer progression. Cancer Res 2011: **71:** 7525**–**7536.

**119** Bhaskar P T, Hay N. The two TORCs and Akt. Dev Cell 2007: **12:** 487-502.

**120** Fang Z, Zhang T, Dizeyi N *et al.* Androgen receptor enhances p27 degradation in prostate cancer cells through rapid and selective TORC2 actviation. J Biol Chem 2012: **287:** 2090-2098.

**121** Wang Y, Mikhailova M, Bose S *et al.* Regulation of androgen receptor transcriptional activity by rapamycin in prostate cancer cell proliferation and survival. Oncogene 2008: **27:** 7106-7117.

**122** [Sertznig P](http://www.ncbi.nlm.nih.gov/pubmed?term=Sertznig P%5BAuthor%5D&cauthor=true&cauthor_uid=20592797), [Seifert M](http://www.ncbi.nlm.nih.gov/pubmed?term=Seifert M%5BAuthor%5D&cauthor=true&cauthor_uid=20592797), [Tilgen W](http://www.ncbi.nlm.nih.gov/pubmed?term=Tilgen W%5BAuthor%5D&cauthor=true&cauthor_uid=20592797) *et al.* Activation of vitamin D receptor (VDR)- and peroxisome proliferator-activated receptor (PPAR)-signaling pathways through 1,25(OH)(2)D(3) in melanoma cell lines and other skin-derived cell lines. Dermatoendocrinol 2009: **1:** 232-238.

**123** [Krämer C](http://www.ncbi.nlm.nih.gov/pubmed?term=Krämer C%5BAuthor%5D&cauthor=true&cauthor_uid=19027855), [Seltmann H](http://www.ncbi.nlm.nih.gov/pubmed?term=Seltmann H%5BAuthor%5D&cauthor=true&cauthor_uid=19027855), [Seifert M](http://www.ncbi.nlm.nih.gov/pubmed?term=Seifert M%5BAuthor%5D&cauthor=true&cauthor_uid=19027855) *et al.* Characterization of the vitamin D endocrine system in human sebocytes in vitro. J Steroid Biochem Mol Biol 2009: **113** :9-16.

**124** [Bikle D D](http://www.ncbi.nlm.nih.gov/pubmed?term=Bikle DD%5BAuthor%5D&cauthor=true&cauthor_uid=21845365). Vitamin D and the skin: Physiology and pathophysiology. Rev Endocr Metab Disord 2012: **13:** 3-19.

**125** Reichrath J. Vitamin D and the skin: an acient friend, revisited. Exp Dermatol 2007: **16:** 618-625.

**126** Reichrath J, Lehmann B, Carlberg C *et al.* Vitamines as hormones. Horm Metab Res 2007: **39:** 71-84.

**127** [An B S](http://www.ncbi.nlm.nih.gov/pubmed?term=An BS%5BAuthor%5D&cauthor=true&cauthor_uid=20733005), [Tavera-Mendoza L E](http://www.ncbi.nlm.nih.gov/pubmed?term=Tavera-Mendoza LE%5BAuthor%5D&cauthor=true&cauthor_uid=20733005), [Dimitrov V](http://www.ncbi.nlm.nih.gov/pubmed?term=Dimitrov V%5BAuthor%5D&cauthor=true&cauthor_uid=20733005) *et al.* Stimulation of Sirt1-regulated FoxO protein function by the ligand-bound vitamin D receptor. Mol Cell Biol 2010: **30:** 4890-4900.

**128** Hayashi N, Watanabe H, Yasukawa H *et al.* Comedolytic effect of

topically appllied vitamin D3 analogue on pseudocomedones in the rhino mouse. Br J Dermatol 2006: **155:** 895-901.

**129** Nieves N J, Ahrens J M, Plum L A *et al.* Identification of a unique subset of 2-methylene-19-nor analogs of vitamin D with comedolytic activity in the rhino mouse. J Invest Dermatol 2010: **130:** 2359-2367.

**130** Ertugrul D T, Karadag A S, Tutal E *et al.* Does isotretinoin have effect on vitamin D physiology and bone metabolism in acne patients? Dermatol Ther 2011: **24:** 291-295.

**131** Nishi K, Yoshida M, Fujiwara D *et al.* Leptomycin B target a regulatory cascade of crm1, a fission yeast nuclear protein, involved in control of higher order chromosome structure and gene expression. J Biol Chem 1994: **269:** 6320-6324.

**132** [Kudo N](http://www.ncbi.nlm.nih.gov/pubmed?term=Kudo N%5BAuthor%5D&cauthor=true&cauthor_uid=9683540), [Wolff B](http://www.ncbi.nlm.nih.gov/pubmed?term=Wolff B%5BAuthor%5D&cauthor=true&cauthor_uid=9683540), [Sekimoto T](http://www.ncbi.nlm.nih.gov/pubmed?term=Sekimoto T%5BAuthor%5D&cauthor=true&cauthor_uid=9683540) *et al.* Leptomycin B inhibition of signal-mediated nuclear export by direct binding to CRM1. Exp Cell Res 1998: **242:** 540-547.

**133** Zhao X, Gan L, Pan H *et al.* Multiple elements regulate nuclear/cytoplasmic shuttling of FOXO1: characterization of phosphorylation- and 14-3-3-dependent and - independent mechanisms. Biochem J 2004: **378:** 839-849.

**134** [Vogt P K](http://www.ncbi.nlm.nih.gov/pubmed?term=Vogt PK%5BAuthor%5D&cauthor=true&cauthor_uid=15917664), [Jiang H](http://www.ncbi.nlm.nih.gov/pubmed?term=Jiang H%5BAuthor%5D&cauthor=true&cauthor_uid=15917664), [Aoki M](http://www.ncbi.nlm.nih.gov/pubmed?term=Aoki M%5BAuthor%5D&cauthor=true&cauthor_uid=15917664). Triple layer control: phosphorylation, acetylation and ubiquitination of FOXO proteins. Cell Cycle 2005: **4:** 908-913.

**135** Jacobs F M, van der Heide L P, Wijchers P J *et al.* Foxo6, a novel member of the FOXO class of transcription factors with distinct shuttling dynamics. J Biol Chem 2003: **278:** 35959-35967.

**136** Brownawell A M, Kops G J, Macara I G *et al.* Inhibition of nuclear import by protein kinase B (Akt) regulates the subcellular distribution and activity of the forkhead transcription factor AFX. Mol Cell Biol 2001: **21:** 3534-3546.

**137** Jang B C, Munoz-Najar U, Paik J H *et al.* Leptomycin B, an inhibitor of the nuclear export receptor CRM1, inhibits COX-2 exppression. J Biol Chem 2003: **278:** 2773-2776.

**138** Kobayashi T, Shinkai H. Leptomycin B reduces matrix metalloproteinase-9 expression and suppresses cutaneous inflammation. J Invest Dermatol 2005: **124:** 331-337.

**139** Kobayashi T. Anti-inflammatory treatment for contact dermatitis by topical leptomycin B: Inhibitory effects on the induced expression of matrix metalloproteinases (MMP)-9 and -3. Eur J Dermatol 2011: **21:** 438-439.

**140** [Alestas T](http://www.ncbi.nlm.nih.gov/pubmed?term=Alestas T%5BAuthor%5D&cauthor=true&cauthor_uid=16388388), [Ganceviciene R](http://www.ncbi.nlm.nih.gov/pubmed?term=Ganceviciene R%5BAuthor%5D&cauthor=true&cauthor_uid=16388388), [Fimmel S](http://www.ncbi.nlm.nih.gov/pubmed?term=Fimmel S%5BAuthor%5D&cauthor=true&cauthor_uid=16388388) *et al.* Enzymes involved in the biosynthesis of leukotriene B4 and prostaglandin E2 are active in sebaceous glands. J Mol Med (Berl) 2006: **84:** 75-87.

**141** Papakonstantinou E, Aletras A J, Glass E *et al.* [Matrix metalloproteinases of epithelial origin in facial sebum of patients with acne and their regulation by isotretinoin.](http://www.ncbi.nlm.nih.gov/pubmed/16185265) J Invest Dermatol 2005: **125:** 673-684.

**142** [Mestre J R](http://www.ncbi.nlm.nih.gov/pubmed?term=Mestre JR%5BAuthor%5D&cauthor=true&cauthor_uid=9067275), [Subbaramaiah K](http://www.ncbi.nlm.nih.gov/pubmed?term=Subbaramaiah K%5BAuthor%5D&cauthor=true&cauthor_uid=9067275), [Sacks P G](http://www.ncbi.nlm.nih.gov/pubmed?term=Sacks PG%5BAuthor%5D&cauthor=true&cauthor_uid=9067275) *et al.* Retinoids suppress phorbol ester-mediated induction of cyclooxygenase-2. Cancer Res 1997: **57:** 1081-1085.

**143** Li H, Liang J, Castrillon D H *et al.* FoxO4 regulates tumor necrosis factor alpha directed smooth muscle cell migration by activating matrix metalloproteinase 9 gene transcription. Mol Cell Biol 2007: **27:** 2676-2686.

**144** Nguyen K T, Holloway M P, Altura R A. The CRM1 nuclear export protein in normal development and disease. Int J Biochem Mol Biol 2012: **3:** 137-151.

**145** Kau T R, Schroeder F, Ramaswamy S *et al.* A chemical genetic screen identifies inhibitors of regulated nuclear export of forkhead transcription factor in PTEN-deficient tumor cells. Cancer Cell 2003: **4:** 463-476.

**146** Turner J G, Dawson J, Sullivan D M. Nuclear export of proteins and drug resistance in cancer. Biochem Pharmacol 2012: **83:** 1021-1032.

**147** Waldmann I, Spillner C, Kehlenbach R H. The nucleoporin-like protein NLP1 (hCG1) promotes CRM1-dependent nuclear protein export. J Cell Sci 2012: **125:** 144-154.

**148** Nguyen K T, Holloway M P, Altura R A. The CRM1 nuclear export protein in normal development and disease. Int J Biochem Mol Biol 2012: **3:** 137-151.

**149** Guerico G, Rivarola M A, Chaler E *et al.* Relationship between the growth hormone/insulin-like growth factor-I axis, insulin sensitivity, and adrenal androgens in normal prepubertal and pubertal girls. J Clin Endocrinol Metab 2003: **88:**1389-1393.

**150** Chen W, Obermayer-Pietsch B, Hong J B *et al.* Acne-associated syndromes: models for better understanding acne pathogenesis. J Eur Acad Dermatol Venereol 2011: **25:** 637-646.

**151** Bergstrom K G. Everything old is new again: spironolactone and metformin in the treatment of acne. J Drugs Dermatol 2010: **9:** 569-571.

**152** Banaszewska B, Pawelczyk L, Spaczynski R Z *et al.* Effects of simvastatin and metformin on polycystic ovary syndrome after six months of treatment. J Clin Endocrinol Metab 2011: **96:** 3493-3501.

**153** Pasquali R, Gambineri A. [Insulin-sensitizing agents in women with polycystic ovary syndrome.](http://www.ncbi.nlm.nih.gov/pubmed/16798283) Fertil Steril 2006: **86** Suppl 1: S28-29.

**154** [Hardie D G](http://www.ncbi.nlm.nih.gov/pubmed?term=Hardie DG%5BAuthor%5D&cauthor=true&cauthor_uid=23102217), [Ross F A](http://www.ncbi.nlm.nih.gov/pubmed?term=Ross FA%5BAuthor%5D&cauthor=true&cauthor_uid=23102217), [Hawley S A](http://www.ncbi.nlm.nih.gov/pubmed?term=Hawley SA%5BAuthor%5D&cauthor=true&cauthor_uid=23102217). AMP-activated protein kinase: a target for drugs both ancient and modern. Chem Biol 2012: **19:** 1222-1236.

**155** [Sinnett-Smith J](http://www.ncbi.nlm.nih.gov/pubmed?term=Sinnett-Smith J%5BAuthor%5D&cauthor=true&cauthor_uid=23159620), [Kisfalvi K](http://www.ncbi.nlm.nih.gov/pubmed?term=Kisfalvi K%5BAuthor%5D&cauthor=true&cauthor_uid=23159620), [Kui R](http://www.ncbi.nlm.nih.gov/pubmed?term=Kui R%5BAuthor%5D&cauthor=true&cauthor_uid=23159620) *et al.* Metformin inhibition of mTORC1 activation, DNA synthesis and proliferation in pancreatic cancer cells: dependence on glucose concentration and role of AMPK. Biochem Biophys Res Commun 2013: **430:** 352-357.

**156** [Sato A](http://www.ncbi.nlm.nih.gov/pubmed?term=Sato A%5BAuthor%5D&cauthor=true&cauthor_uid=23197693), [Sunayama J](http://www.ncbi.nlm.nih.gov/pubmed?term=Sunayama J%5BAuthor%5D&cauthor=true&cauthor_uid=23197693), [Okada M](http://www.ncbi.nlm.nih.gov/pubmed?term=Okada M%5BAuthor%5D&cauthor=true&cauthor_uid=23197693) *et al.* Glioma-initiating cell elimination by metformin activation of FOXO3 via AMPK. Stem Cells Transl Med 2012: **1:** 811-824.

**157** Greer E L, Oskoui P R, Banko M R *et al.* The energy sensor AMP-activated protein kinase directly regulates the mammalian FOXO3 transcription factor. J Biol Chem 2007: **282:** 30107-30119.

**158** Khatri S, Yepiskoposyan H, Gallo C A *et al.* FoxO3a regulates glycolysis via transcriptional control of tumor suppressor TSC1. J Biol Chem 2010: **285:** 15960-15965.

**159** Chen C C, Jeon S M, Bhaskar P T *et al.* FoxOs inhibit mTORC1 and activate Akt by inducing the expression of Sestrin3 and Rictor. Dev Cell 2010: **18:** 592-604.

**160** [Feldman M E](http://www.ncbi.nlm.nih.gov/pubmed?term=Feldman ME%5BAuthor%5D&cauthor=true&cauthor_uid=20549474), [Shokat K M](http://www.ncbi.nlm.nih.gov/pubmed?term=Shokat KM%5BAuthor%5D&cauthor=true&cauthor_uid=20549474). New inhibitors of the PI3K-Akt-mTOR pathway: insights into mTOR signaling from a new generation of Tor kinase domain inhibitors (TORKinibs). Curr Top Microbiol Immunol 2010: **347:** 241-262.

**161** Inoki K, Zhu T, Guan K L. TSC2 mediates cellular energy response to control cell growth and survival. Cell 2003: **115:** 577-590.

**162** Gwinn D M, Shackelford D B, Egan D F *et al.* AMPK phosphorylation of raptor mediates a metabolic checkpoint. Mol Cell 2008: **30:** 214-226.

**163** [Park C S](http://www.ncbi.nlm.nih.gov/pubmed?term=Park CS%5BAuthor%5D&cauthor=true&cauthor_uid=23041647), [Bang B R](http://www.ncbi.nlm.nih.gov/pubmed?term=Bang BR%5BAuthor%5D&cauthor=true&cauthor_uid=23041647), [Kwon H S](http://www.ncbi.nlm.nih.gov/pubmed?term=Kwon HS%5BAuthor%5D&cauthor=true&cauthor_uid=23041647) *et al.* Metformin reduces airway inflammation and remodeling via activation of AMP-activated protein kinase. Biochem Pharmacol 2012: **84:** 1660-1670.

**164** [Bikman B T](http://www.ncbi.nlm.nih.gov/pubmed?term=Bikman BT%5BAuthor%5D&cauthor=true&cauthor_uid=20798864), [Zheng D](http://www.ncbi.nlm.nih.gov/pubmed?term=Zheng D%5BAuthor%5D&cauthor=true&cauthor_uid=20798864), [Kane D A](http://www.ncbi.nlm.nih.gov/pubmed?term=Kane DA%5BAuthor%5D&cauthor=true&cauthor_uid=20798864) *et al.* Metformin improves insulin signaling in obese rats via reduced IKKbeta action in a fiber-type specific manner. J Obes 2010: **2010:** pii: 970865

**165** [Weichhart T](http://www.ncbi.nlm.nih.gov/pubmed?term=Weichhart T%5BAuthor%5D&cauthor=true&cauthor_uid=18848473), [Costantino G](http://www.ncbi.nlm.nih.gov/pubmed?term=Costantino G%5BAuthor%5D&cauthor=true&cauthor_uid=18848473), [Poglitsch M](http://www.ncbi.nlm.nih.gov/pubmed?term=Poglitsch M%5BAuthor%5D&cauthor=true&cauthor_uid=18848473) *et al.* The TSC-mTOR signaling pathway regulates the innate inflammatory response. Immunity 2008: **29:** 565-577.

**166** Kalender A, Selvaraj A, Kim S Y *et al.* Metformin, independent of AMPK, inhibits mTORC1 in a Rag GTPase-dependent manner. Cell Metab 2010: **11:** 390-401.

**167** Marques F Z, Markus M A, Morris B J. Resveratrol: cellular actions of a potent natural chemical that confers a diversity of health benefits. Int J Biochem Cell Biol 2009: **41:** 2125-2128.

**168** Zhou H, Luo Y, Huang S. Updates of mTOR inhibitors. Anticancer Agents Med Chem 2010: **10:** 571-581.

**169** Jiang H, Shang X, Wu H *et al.* Resveratrol downregulates PI3K/Akt/mTOR signaling pathways in human U251 glioma cells. J Exp Ther Oncol 2009: **8:** 25-33.

**170** Brito P M, Devillard R, Negre-Salvayre A *et al.* Resveratrol inhibits the mTOR mitogenic signaling evoked by oxidized LDL in smooth muscle cells. Atherosclerosis 2009: **205:** 126-134.

**171** Lin J N, Lin V C, Rau K M *et al.* Resveratrol modulates tumor cell proliferation and protein translation via SIRT1-dependent AMPK activation. J Agric Food Chem 2010: **58:** 1584-1592.

**172** Fröjdö S, Cozzone D, Vidal H *et al.* Resveratrol is a class IA phosphoinositide 3-kinase inhibitor. Biochem J 2007: **406:** 511-518.

**173**  [Villa-Cuesta E](http://www.ncbi.nlm.nih.gov/pubmed?term=Villa-Cuesta E%5BAuthor%5D&cauthor=true&cauthor_uid=22242130), [Boylan J M](http://www.ncbi.nlm.nih.gov/pubmed?term=Boylan JM%5BAuthor%5D&cauthor=true&cauthor_uid=22242130), [Tatar M](http://www.ncbi.nlm.nih.gov/pubmed?term=Tatar M%5BAuthor%5D&cauthor=true&cauthor_uid=22242130) *et al.* Resveratrol inhibits protein translation in hepatic cells. PLoS One 2011: **6:** e29513.

**174** Fabbrocini G, Staibano S, De Rosa G*et al.* [Resveratrol-containing gel for the treatment of acne vulgaris: a single-blind, vehicle-controlled, pilot study.](http://www.ncbi.nlm.nih.gov/pubmed/21348544) Am J Clin Dermatol 2011: **12:** 133-141.

**175** [Docherty J J](http://www.ncbi.nlm.nih.gov/pubmed?term="Docherty JJ"%5BAuthor%5D), [McEwen H A](http://www.ncbi.nlm.nih.gov/pubmed?term="McEwen HA"%5BAuthor%5D), [Sweet T J](http://www.ncbi.nlm.nih.gov/pubmed?term="Sweet TJ"%5BAuthor%5D) *et al.* Resveratrol inhibition of Propionibacterium acnes. J Antimicrob Chemother 2007: **59:** 1182-1184.

**176** [Coenye T](http://www.ncbi.nlm.nih.gov/pubmed?term=Coenye T%5BAuthor%5D&cauthor=true&cauthor_uid=22305279), [Brackman G](http://www.ncbi.nlm.nih.gov/pubmed?term=Brackman G%5BAuthor%5D&cauthor=true&cauthor_uid=22305279), [Rigole P](http://www.ncbi.nlm.nih.gov/pubmed?term=Rigole P%5BAuthor%5D&cauthor=true&cauthor_uid=22305279) *et al.* Eradication of Propionibacterium acnes biofilms by plant extracts and putative identification of icariin, resveratrol and salidroside as active compounds. Phytomedicine 2012: **19:** 409-412.

**177** [Simonart T](http://www.ncbi.nlm.nih.gov/pubmed?term=Simonart T%5BAuthor%5D&cauthor=true&cauthor_uid=22920095). Newer approaches to the treatment of acne vulgaris. Am J Clin Dermatol 2012: **13:** 357-364.

**178** [Reuter J](http://www.ncbi.nlm.nih.gov/pubmed?term="Reuter J"%5BAuthor%5D), [Wölfle U](http://www.ncbi.nlm.nih.gov/pubmed?term="Wölfle U"%5BAuthor%5D), [Weckesser S](http://www.ncbi.nlm.nih.gov/pubmed?term="Weckesser S"%5BAuthor%5D) *et al.* Which plant for which skin disease? Part 1: Atopic dermatitis, psoriasis, acne, condyloma and herpes simplex. J Dtsch Dermatol Ges 2010: **8:** 788-796.

**179** [Fowler J F Jr](http://www.ncbi.nlm.nih.gov/pubmed?term="Fowler JF Jr"%5BAuthor%5D), [Woolery-Lloyd H](http://www.ncbi.nlm.nih.gov/pubmed?term="Woolery-Lloyd H"%5BAuthor%5D), [Waldorf H](http://www.ncbi.nlm.nih.gov/pubmed?term="Waldorf H"%5BAuthor%5D) *et al*. Innovations in natural ingredients and their use in skin care. J Drugs Dermatol 2010: **9** (6 Suppl): S72-S81.

**180** [Reuter J](http://www.ncbi.nlm.nih.gov/pubmed?term="Reuter J"%5BAuthor%5D), [Merfort I](http://www.ncbi.nlm.nih.gov/pubmed?term="Merfort I"%5BAuthor%5D), [Schempp C M](http://www.ncbi.nlm.nih.gov/pubmed?term="Schempp CM"%5BAuthor%5D). Botanicals in dermatology: an evidence-based review. Am J Clin Dermatol 2010: **11:** 247-267.

**181** [Liao S](http://www.ncbi.nlm.nih.gov/pubmed?term="Liao S"%5BAuthor%5D). The medicinal action of androgens and green tea epigallocatechin gallate. Hong Kong Med J 2001: **7:** 369-374.

**182** [Elsaie M L](http://www.ncbi.nlm.nih.gov/pubmed?term="Elsaie ML"%5BAuthor%5D), [Abdelhamid M F](http://www.ncbi.nlm.nih.gov/pubmed?term="Abdelhamid MF"%5BAuthor%5D), [Elsaaiee L T](http://www.ncbi.nlm.nih.gov/pubmed?term="Elsaaiee LT"%5BAuthor%5D) *et al.* The efficacy of topical 2% green tea lotion in mild-to-moderate acne vulgaris. J Drugs Dermatol 2009: **8:** 358-364.

**183** [Mahmood T](http://www.ncbi.nlm.nih.gov/pubmed?term="Mahmood T"%5BAuthor%5D), [Akhtar N](http://www.ncbi.nlm.nih.gov/pubmed?term="Akhtar N"%5BAuthor%5D), [Khan B A](http://www.ncbi.nlm.nih.gov/pubmed?term="Khan BA"%5BAuthor%5D) *et al.* Outcomes of 3% green tea emulsion on skin sebum production in male volunteers. Bosn J Basic Med Sci 2010: **10:** 260-264.

**184** [Im M](http://www.ncbi.nlm.nih.gov/pubmed?term=Im M%5BAuthor%5D&cauthor=true&cauthor_uid=22763784), [Kim S Y](http://www.ncbi.nlm.nih.gov/pubmed?term=Kim SY%5BAuthor%5D&cauthor=true&cauthor_uid=22763784), [Sohn K C](http://www.ncbi.nlm.nih.gov/pubmed?term=Sohn KC%5BAuthor%5D&cauthor=true&cauthor_uid=22763784) *et al*. Epigallocatechin-3-gallate suppresses IGF-I-induced lipogenesis and cytokine expression in SZ95 sebocytes. J Invest Dermatol 2012: **132:** 2700-2708.

**185** Yoon J Y, Kwon H H, Min S U *et al.* Epigallocatechin-3-gallate improves acne in humans by modulating intracellular targets and inhibiting P. acnes. J Invest Dermatol 2013: **133:** 429-440.

**186** Van Aller G S, Carson J D, Tang W *et al.* Epigallocatechin gallate (EGCG), a major component of green tea, is a dual phosphoinositide-3-kinase/mTOR inhibitor. Biochem Biophys Res Commun 2011: **406:** 194-199.

**187** Huang C H, Tsai S J, Wang Y J *et al*. EGCG inhibits protein synthesis, lipogenesis, and cell cycle progression through activation of AMPK in p53 positive and negative human hepatoma cells. Mol Nutr Food Res 2009: **53:** 1156-1165.

**188** Bartholome A, Kampkötter A, Tanner S *et al*. Epigallocatechin gallate-induced modulation of FoxO signaling in mammalian cells and C. elegans: FoxO stimulation is masked via PI3K/Akt activation by hydrogen peroxide formed in cell culture. Arch Biochem Biophys 2010: **501:** 58-64.

**189** Lin Y G, Kunnumakkara A B, Nair A *et al.* Curcumin inhibits tumor growth

and angiogenesis in ovarian carcinoma by targeting the nuclear factor-kappaB pathway. Clin Cancer Res 2007: **13:** 3423-3430.

**190** Goel A, Kunnumakkara A B, Aggarwal B B. Curcumin as “Curecumin”: from kitchen to clinic. Biochem Pharmacol 2008: **75:** 787-809.

**191**  Johnson S M, Gulhati P, Arrieta I *et al.* Curcumin inhibits proliferation of colorectal carcinoma by modulating Akt/mTOR signaling. Anticancer Res

2009: **29:** 3185-3190.

**192** Beevers C S, Li F, Liu L *et al.* Curcumin inhibits the mammalian target of rapamycin-mediated signaling pathways in cancer cells. Int J Cancer 2006: **119:** 757-764.

**193** Beevers C S, Chen L, Liu L *et al.* Curcumin disrupts the mammalian target of rapamycin-raptor complex. Cancer Res 2009: **69:** 1000-1008.

**194** [Liu C H](http://www.ncbi.nlm.nih.gov/pubmed?term=Liu CH%5BAuthor%5D&cauthor=true&cauthor_uid=22976319), [Huang H Y](http://www.ncbi.nlm.nih.gov/pubmed?term=Huang HY%5BAuthor%5D&cauthor=true&cauthor_uid=22976319). Antimicrobial activity of curcumin-loaded myristic acid microemulsions against Staphylococcus epidermidis. Chem Pharm Bull (Tokyo) 2012: **60:** 1118-1124.

**195** [Banerjee S](http://www.ncbi.nlm.nih.gov/pubmed?term="Banerjee S"%5BAuthor%5D), [Kong D](http://www.ncbi.nlm.nih.gov/pubmed?term="Kong D"%5BAuthor%5D), [Wang Z](http://www.ncbi.nlm.nih.gov/pubmed?term="Wang Z"%5BAuthor%5D) *et al.* Attenuation of multi-targeted proliferation-linked signaling by 3,3'-diindolylmethane (DIM): from bench to clinic. Mutat Res 2011: **728:** 47-66.

**196** Kong D, Banerjee S, Huang W *et al.* [Mammalian target of rapamycin repression by 3,3'-diindolylmethane inhibits invasion and angiogenesis in platelet-derived growth factor-D-overexpressing PC3 cells.](http://www.ncbi.nlm.nih.gov/pubmed/18339874) Cancer Res 2008: **68:** 1927-1934.

**197** [Eto I](http://www.ncbi.nlm.nih.gov/pubmed?term="Eto I"%5BAuthor%5D). Nutritional and chemopreventive anti-cancer agents up-regulate expression of p27Kip1, a cyclin-dependent kinase inhibitor, in mouse JB6 epidermal and human MCF7, MDA-MB-321 and AU565 breast cancer cells. Cancer Cell Int 2006: **6:** 20.

**198** Foukas L C, Daniele N, Ktori C *et al.* Direct effects of caffeine and theophylline on p110delta and other phosphoinositide 3-kinases. Differential effects on lipid kinase and protein kinase activities. J Biol Chem 2002: **277:** 37124-37130.

**199** Kudchodkar S B, Yu Y, Maguire T G *et al.* Human cytomegalovirus infection alters the substrate specificities and rapamycin sensitivities of raptor- and rictor-containing complexes. Proc Natl Acad Sci USA 2006: **103:** 14182-14187.

**200** [Saiki S](http://www.ncbi.nlm.nih.gov/pubmed?term="Saiki S"%5BAuthor%5D), [Sasazawa Y](http://www.ncbi.nlm.nih.gov/pubmed?term="Sasazawa Y"%5BAuthor%5D), [Imamichi Y](http://www.ncbi.nlm.nih.gov/pubmed?term="Imamichi Y"%5BAuthor%5D) *et al.* Caffeine induces apoptosis by enhancement of autophagy via PI3K/Akt/mTOR/p70S6K inhibition. Autophagy 2011: **7:** 176-187.

**201** Vaid M, Katiyar S K. Molecular mechanisms of inhibition of photocarcinogenesis by silymarin, a phytochemical from milk thistle(Silybum marianum L. Gaertn). Int J Oncol 2010: **36:** 1053-1060.

**202** Nichols J A, Katiyar S K. Skin photoprotection by natural polyphenols: anti-inflammatory, anti-oxidant and DNA repair mechanisms. Arch Dermatol Res 2010: **302:** 71.

**203** Gharagozloo M, Javid E N, Rezaei A *et al.* [Silymarin inhibits cell cycle progression and mTOR activity in activated human T cells: therapeutic implications for autoimmune diseases.](http://www.ncbi.nlm.nih.gov/pubmed/23121838) Basic Clin Pharmacol Toxicol 2013: **112:** 251-256.

**204** [Zhou H](http://www.ncbi.nlm.nih.gov/pubmed?term=Zhou H%5BAuthor%5D&cauthor=true&cauthor_uid=20812900), [Luo Y](http://www.ncbi.nlm.nih.gov/pubmed?term=Luo Y%5BAuthor%5D&cauthor=true&cauthor_uid=20812900), [Huang S](http://www.ncbi.nlm.nih.gov/pubmed?term=Huang S%5BAuthor%5D&cauthor=true&cauthor_uid=20812900). Updates of mTOR inhibitors. Anticancer Agents Med Chem 2010: **10:** 571-581.

**205** [Feldman M E](http://www.ncbi.nlm.nih.gov/pubmed?term=Feldman ME%5BAuthor%5D&cauthor=true&cauthor_uid=20549474), [Shokat K M](http://www.ncbi.nlm.nih.gov/pubmed?term=Shokat KM%5BAuthor%5D&cauthor=true&cauthor_uid=20549474). New inhibitors of the PI3K-Akt-mTOR pathway: insights into mTOR signaling from a new generation of Tor kinase domain inhibitors (TORKinibs). Curr Top Microbiol Immunol 2010: **347:** 241-262.

**206** Liu Q, Kang S A, Thoreen C C *et al.* [Development of ATP-competitive mTOR inhibitors.](http://www.ncbi.nlm.nih.gov/pubmed/22125084) Methods Mol Biol 2012: **821:** 447-460.

**207** Lisse T S, Hewison M. Vitamin D. A new player in the world of mTOR signaling. Cell Cycle 2011: **10:12:** 1888-1889.

**208** Lisse T S, Liu T, Irmler M *et al*. Gene targeting by the vitamin D response element binding protein reveals a role for vitamin D in osteoblast mTOR signaling. FASEB J 2011: **25:** 937-947.

**209** Sutherland E R, Goleva E, Leisa P *et al.* Vitamin D levels, lung function, and steroid response in adult asthma. Am J Respir Crit Care Med 2010: **181**: 699-704.

**210** Zhang Y, Leung D Y, Richers B N *et al.* Vitamin D inhibits monocyte/ macrophage proinflammatory cytokine production by targeting MAPK phosphatase-1. J Immunol 2012: **188:** 2127-2135.

**211** Griffin A C 3^rd^, Kern M J, Kirkwood K L. MKP-1 is essential for canonical vitamin-induced signaling through nuclear import and regulates RANKL expression and function. Mol Endocrinol 2012: **26:** 1682-1693.

**212** Wang X, Liu Y. Regulation of innate immune response by MAP kinase phosphatase-1. Cell Signal 2007: **19:** 1372-1382.

**213** Wancket L M, Frazier W J, Liu Y. Mitogen-activated protein kinase phosphatase (MKP)-1 in immunology, physiology, and disease. Life Sci 2012: **90:** 237-248.

**214** Huang G, Chi L Z, Chi H. Regulation of JNK and p38 MAPK in the immune system: signal integration, propagation and termination. Cytokine 2009: **48:** 161-169.

**215** Maguire O, Pollock C, Martin P *et al.* Regulation of CYP3A4 and CYP3A5 expression and modulation of „intracrine“ metabolism of androgens in prostate cells by liganded vitamin D receptor. Mol Cell Endocrinol 2012, **364:** 54-64.

**216** Melnik B C, Zouboulis C C. Potential role of FoxO1 and mTORC1 in the pathogenesis of Western diet-induced acne. Exp Dermatol 2013: **22:** 311-315.

**217** Melnik B C. Western diet-mediated mTORC1-signaling in acne, psoriasis, atopic dermatitis, and related diseases of civilization: Therapeutic role of plant-derived natural mTORC1 inhibitors. In: Watson R R, Zibadi S (eds). Bioactive dietary factors and plant extracts in dermatology. Springer Science-Business Media New York, 2013, chapt 37, pp.397-420.

**218** Melnik B C. Acne and diet. Hautarzt **64:** 252-262.

**219** Burris J, Rietkerk W, Woolf K. Acne: the role of medical nutrition therapy. J Acad Nutr Diet 2013: **113:** 416-430.

**Figure legends**

**Figure S1. FoxO shuttling between nucleus and cytoplasm:** Increased insulin/IGF-1 signaling (IIS) of puberty superimposed upon by high IIS of Western diet activates the kinase Akt. Akt phosphorylates nuclear FoxO1 protein and promotes FoxO1 nuclear export, thus suppressing FoxO1-mediated target gene regulation.

**Figure S2. Drug-mediated modifications of nuclear FoxO trafficking:** Potential effects of isotretinoin, *all-trans* retinoic acid (ATRA), doxycycline (Doxy) and benzoyl peroxide (BPO) on upregulation of nuclear FoxO1 levels. Isotretinoin after isomerization to ATRA induces the expression of FoxO3, which promotes the expression of FoxO1. ATRA via induction of STRA8 may interfere with CRM1-mediated nuclear FoxO1 export. Doxycycline inhibits the expression of CRM1, thus may enhance nuclear FoxO1 levels. BPO increases cellular levels of reactive oxygen species (ROS) activating ROS-sensing kinases JNK and MST1, which increase nuclear FoxO1 import. Thus, all commonly used anti-acne drugs may enhance FoxO1-mediated gene regulation.

**Figure S3. Inhibition of mTORC1 activity by anti-acne agents:** Oral isotretinoin, *all-trans* retinoic acid (ATRA), doxycycline (Doxy), and benzoyl peroxide (BPO) increase nuclear FoxO levels, which increase the expression of Sestrin3. Sestrin3 activates AMPK and augments the inhibitory function of TSC2 towards Rheb, thus suppressing mTORC1. BPO may stimulate ROS-mediated activation of ATM, a further stimulator of AMPK-mediated mTORC1 inhibition. Azelaic acid (AzA) via inhibition of mitochondrial respiration may increase ROS-mediated upregulation of FoxOs and FoxO-induced Sestrin3 as well as cellular AMP levels, activating AMPK, as does metformin, a well known activator of AMPK. Furthermore, metformin inhibits the Rag/Ragulator-mediated amino acid (AA)-dependent activation of mTORC1. Antiandrogens inhibit mTORC2-dependent activation of Akt, thus increasing TSC1/TSC2-mediated inhibition of Rheb. Antiandrogens suppress the expression of L-type amino acids transporter (LAT), thus interfering with AA-mediated activation of mTORC1. Natural mTORC1 inhibitors like resveratrol and epigallocatechin-3-gallate (EGCG) as well as synthetic mTOR inhibitors inhibit the ATP-dependent kinase activity of mTOR, thereby directly reducing mTORC1 activity. Vitamin D activates the expression of DNA damage-inducible transcript 4 (DDIT4), which activates TSC2 inhibitory function towards mTORC1. Thus, all anti-acne drugs directly or indirectly impair downstream mTORC1 signaling and attenuate cell growth, proliferation and lipogenesis. A Paleolithic diet, which reduces enhanced insulin/IGF1 signaling (IIS) of Western diet due to high glycaemic load and dairy consumption, thus exerts synergistic effects with pharmacologic agents in the treatment of acne.

**Abbreviations**

Akt=Akt kinase (protein kinase B)

AMP=adenosine monophosphate

AMPK=AMP-activated protein kinase

AR=androgen receptor

ATP=adenosine triphosphate

ATRA=*all-trans*-retinoic acid

BPO=benzoyl peroxide

COX=cyclooxygenase

CRM1=chromosomal region maintenance protein 1 (=exportin-1)

1,25D3=1,25-dihydroxyvitamin D3

DDIT4=DNA damage-inducible transcript 4

EGCG= epigallocatechin-3-gallate

FoxO= forkhead box class O transcription factor

4E-BP= eukaryotic initiation factor (eIF) 4E-binding protein

IGF=insulin-like growth factor

IGF1R=IGF-1 receptor

IKKbeta=inhibitor of kappa light chain gene enhancer in B cells

IL=interleukin

IRS=insulin receptor substrate

JNK=Jun-N-terminus kinase

LKB=liver kinase B

LMB=leptomycin B

LXR=liver X receptor

MKP-1=MAPK phosphatase-1

MMP=matrix metalloproteinase

MST1=STE20-like protein kinase-1

mTOR=mechanistic (mammalian) target of rapamycin

NES=nuclear export signal

NLS=nuclear localization signal

NF-κB=nuclear factor kappa B

P. acnes=Propionibacterium acnes

PAK=p21-activated kinase

PCOS=polycystic ovary syndrome

PI3K=phosphoinositol-3 kinase

Rag=Ras-related GTP-binding protein

Raptor=regulatory associated protein of mTOR

RAR=retinoic acid receptor

RXR=retinoid X receptor

Rheb=Ras homolog enriched in brain

Rictor=rapamycin-insensitive companion of mTOR

RXR=retinoid X receptor

ROS=reactive oxygen species

SG=sebaceous gland

S6K=p70 S6 kinase

SREBP=sterol regulatory element binding protein

STRA8=stimulated by retinoic acid 8

TOR=target of rapamycin

TSC=tuberous sclerosis complex

TSC1=hamartin

TSC2=tuberin

VDR=vitamin D receptor

**Table S1.** Action of commonly used* and potential new anti-acne drugs correcting imbalanced FoxO1/mTORC1 signaling in acne

| Isotretinioin* (ATRA) | ATRA🡹, FoxO3🡹, FoxO1🡹 | mTORC1🡻 |
| --- | --- | --- |
| All-trans retinoic acid* (ATRA) | FoxO3🡹, FoxO1🡹  STRA8🡹, CRM1-FoxO1🡹  | mTORC1🡻 |
| Benzoyl peroxide* (BPO) | ROS🡹, JNK🡹, MST1🡹, FoxOs🡹 Sestrin3🡹, AMPK🡹, TSC2🡹  ROS🡹, ATM🡹, AMPK🡹, TSC2🡹 | mTORC1🡻 |
| Doxycycline* | CRM1🡻, FoxO1🡹 | mTORC1🡻 |
| Leptomycin? | CRM1🡻, FoxO1🡹 | mTORC1🡻 |
| Metformin | AMPK🡹, TSC2🡹  Leucine-Rag-Ragulator🡻 | mTORC1🡻 |
| Resveratrol | PI3K🡻, Akt🡻, TSC2🡹,TOR kinase🡻 | mTORC1🡻 |
| Epigallocatechin-3-gallate (EGCG) | AMPK🡹, TSC2🡹  PI3K🡻, Akt🡻, TSC2🡹 | mTORC1🡻 |
| Rapamycin (Sirolimus)?  Rapalogs (Everolimus)? | Rapamycin-FKB12-complex🡹  Rapalog-FKB12-complex🡹 | mTORC1🡻 |
| Synthetic TOR kinase inhibitors (TORkinibs)? | TOR kinase🡻 | mTORC1🡻 |
| Antiandrogens* | mTORC2🡻, Akt🡻, FoxO1🡹  mTORC2🡻, Akt🡻, TSC2🡹  LAT🡻, Leucine-Rag/Ragulator🡻 | mTORC1🡻 |
| Vitamin D (analogs)? | DDIT4🡹, TSC1/TSC2🡹 | mTORC1🡻 |
| Azelaic acid* (AzA) | Mitochondrial respiration 🡻 ROS🡹, FoxO🡹, Sestrin3🡹, AMPK🡹, TSC2🡹  ATP🡻, AMPK🡹, TSC2🡹 | mTORC1🡻 |
| Erythromycin* and other macrolides | ERK1/2🡻, TSC2🡹  TNFα🡻, IKKβ🡻, TSC1🡹        | mTORC1🡻 |
