# Supplementary material for: Are therapeutic effects of antiacne agents mediated by activation of FoxO1 and inhibition of mTORC1?
Source: Exp Dermatol. 2013 Jun 25;22(7):502–4. doi: 10.1111/exd.12172 (PMC3746104; doi:10.1111/exd.12172)
Supplement: Supplementary file 2 [file exd0022-0502-SD2.ppt]

## Slide 1
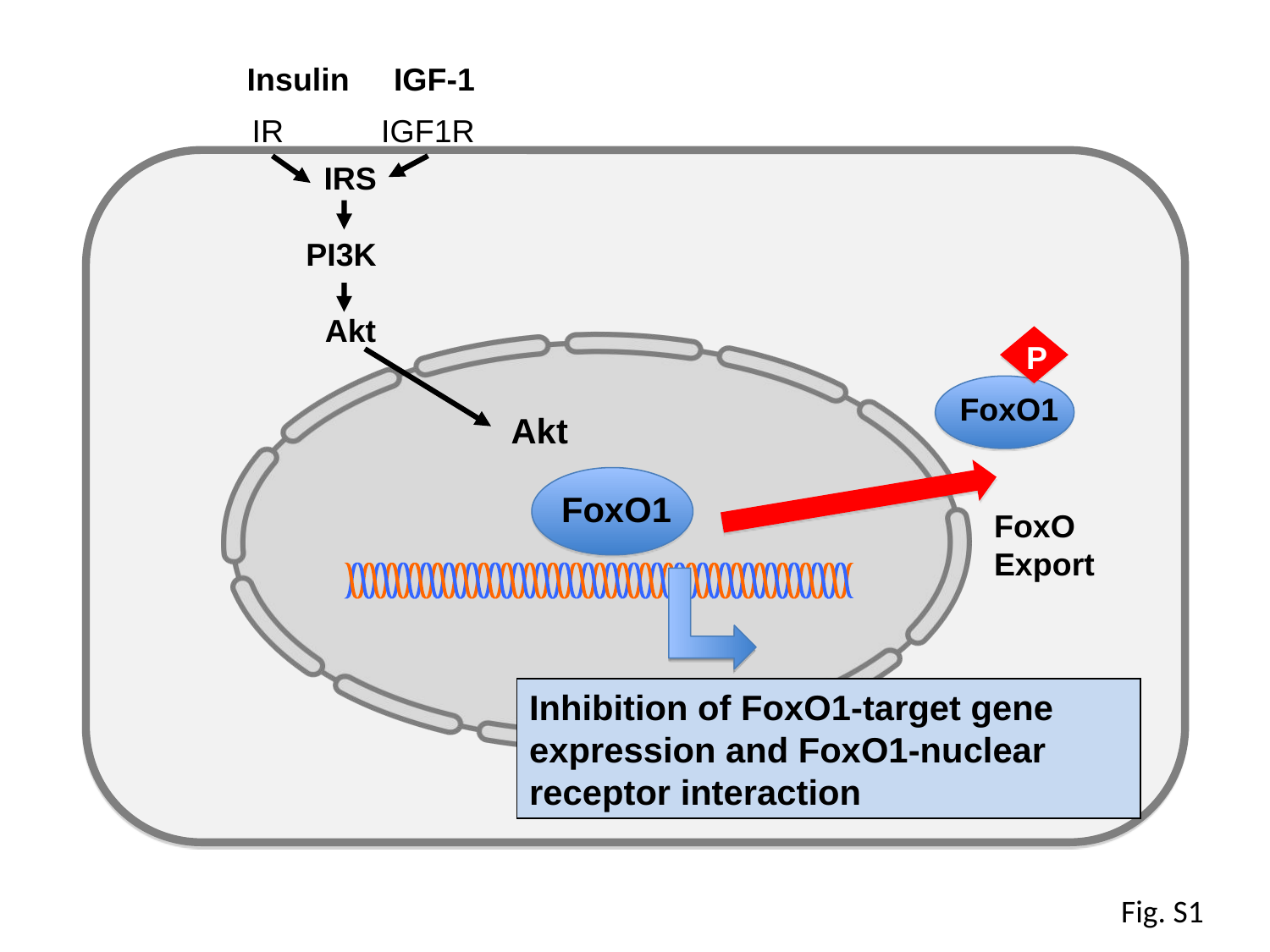

Insulin IGF-1
 IR IGF1R
 IRS
 PI3K
	 Akt
P
 P
FoxO1
Akt
FoxO1
FoxO
Export
Inhibition of FoxO1-target gene expression and FoxO1-nuclear receptor interaction
Fig. S1

## Slide 2
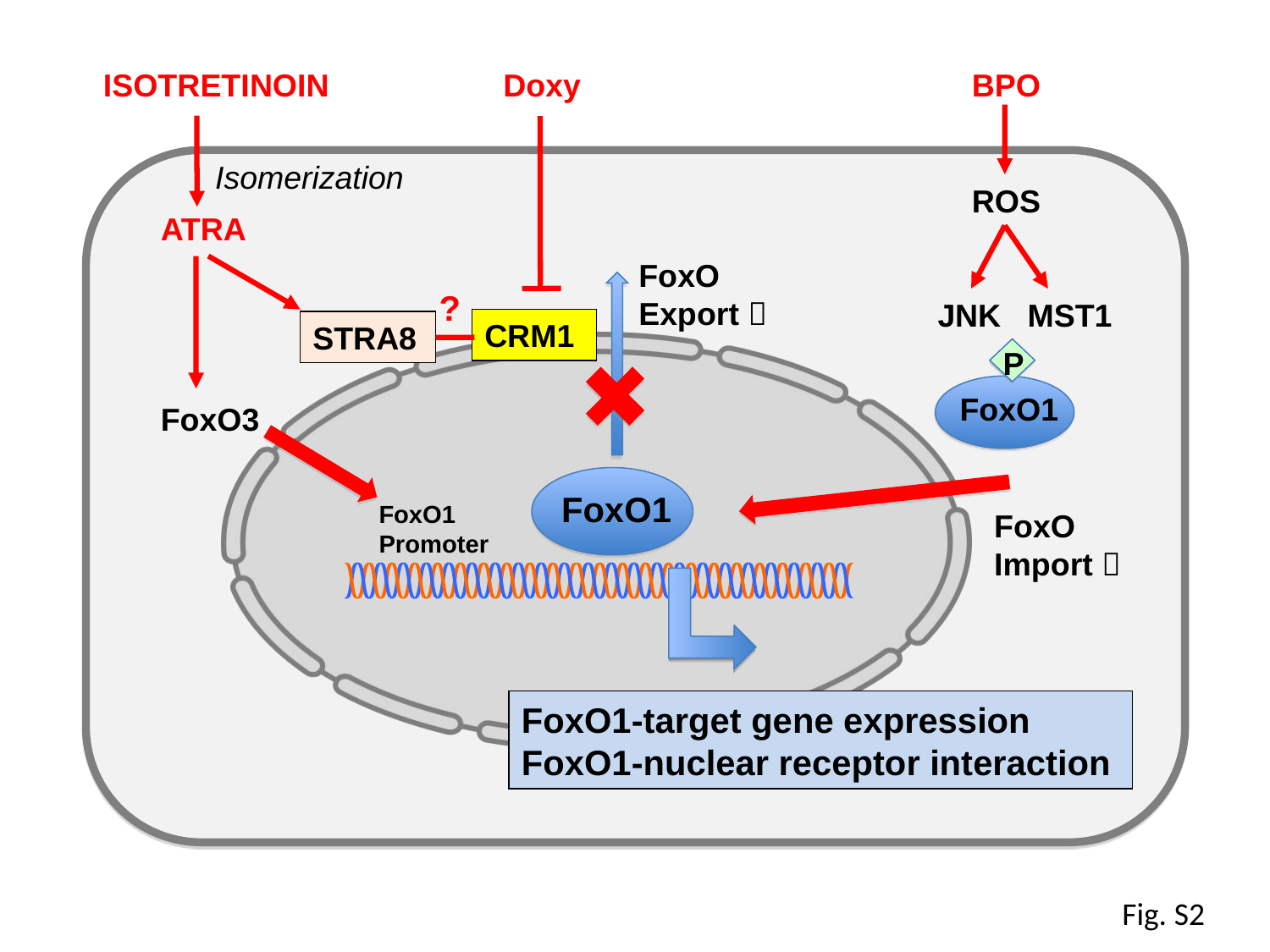

ISOTRETINOIN 	 Doxy BPO
Isomerization
 ATRA
 FoxO3
 ROS
FoxO
Export 
?
JNK MST1
CRM1
STRA8
 P
FoxO1
FoxO1
FoxO1
Promoter
FoxO
Import 
FoxO1-target gene expression
FoxO1-nuclear receptor interaction
Fig. S2

## Slide 3
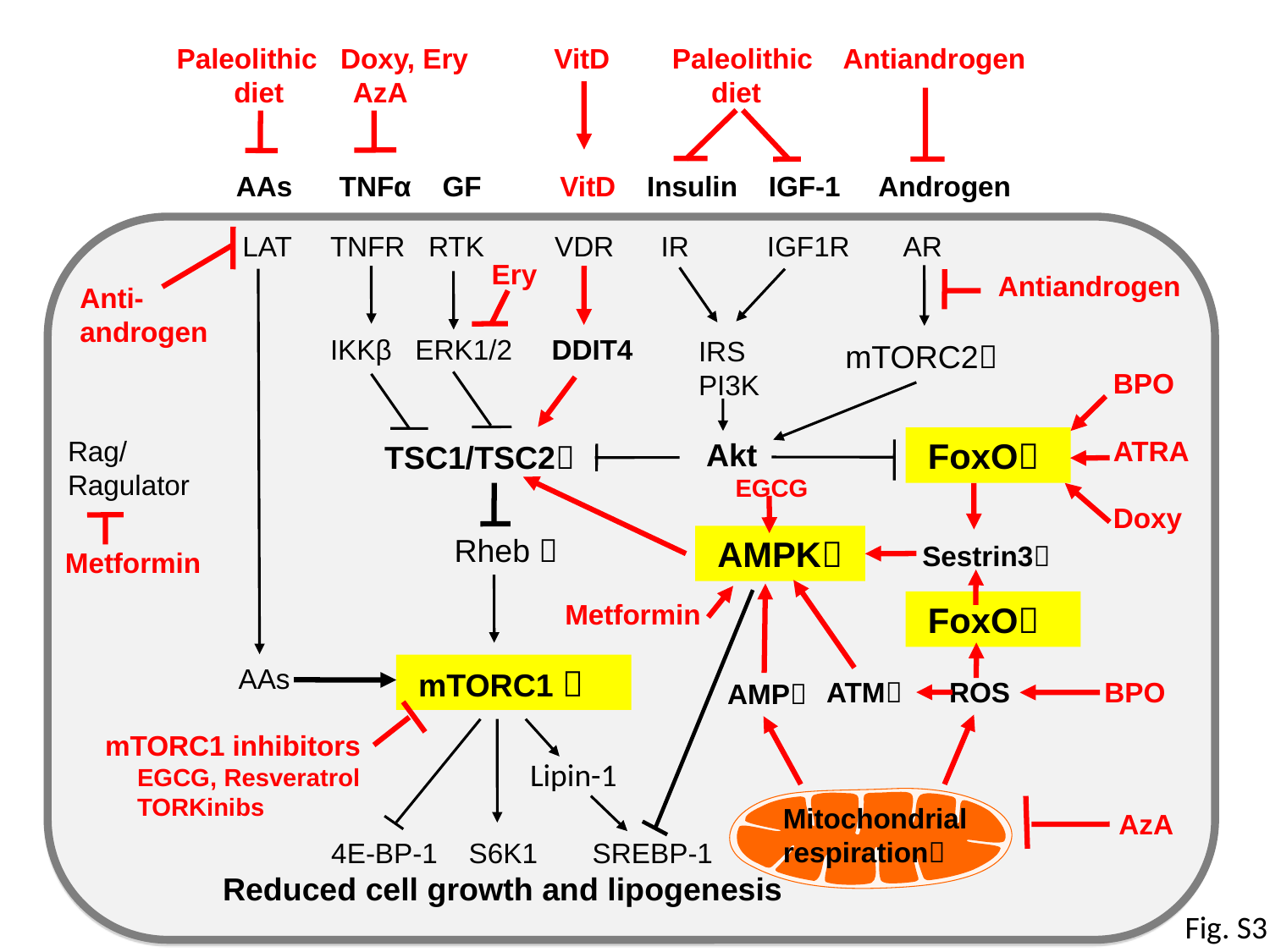

Paleolithic Doxy, Ery VitD Paleolithic Antiandrogen
 diet AzA diet
 AAs TNFα GF VitD Insulin IGF-1 Androgen
 LAT TNFR RTK VDR IR IGF1R AR
Ery
Antiandrogen
Anti-
androgen
IKKβ ERK1/2 DDIT4
IRS
PI3K
 Akt
mTORC2
BPO
ATRA
Doxy
Rag/
Ragulator
 FoxO
 TSC1/TSC2
EGCG
Rheb 
 Sestrin3
 AMPK
Metformin
Metformin
 FoxO
 AAs
 mTORC1 
 AMP
ATM ROS BPO
 mTORC1 inhibitors
	EGCG, Resveratrol
	TORKinibs
 Lipin-1
Mitochondrial
respiration
 AzA
 4E-BP-1 S6K1 SREBP-1
 Reduced cell growth and lipogenesis
Fig. S3
